# A Stacking Ensemble-Based Framework for Predicting the Compressive Strength of Microwave-Cured Geopolymers

**DOI:** 10.3390/ma19122474

**Published:** 2026-06-09

**Authors:** Sarah Nahd Kadhim, Ahmet Emin Kurtoğlu, Derya Bakbak, Abdulkadir Çevik, Ali İhsan Özçetin

**Affiliations:** 1 Department of Civil Engineering, Gaziantep University, Gaziantep 27310, Türkiye; sarahkadhim93@gmail.com (S.N.K.); akcevik@gantep.edu.tr (A.Ç.); 2 Department of Civil Engineering, Iğdır University, Iğdır 76000, Türkiye; aemin.kurtoglu@igdir.edu.tr; 3 Grand National Assembly of Türkiye (TBMM), Ankara 06543, Türkiye; derya.bakbak@tbmm.gov.tr; 4 Şahinbey Municipality, Şahinbey, Gaziantep 27070, Türkiye

**Keywords:** compressive strength, geopolymer, machine learning, microwave curing, stacking ensemble, sustainable construction

## Abstract

Microwave curing offers energy-efficient geopolymer synthesis, yet optimizing compressive strength remains challenging due to complex variable interactions. This study develops an interpretable stacking ensemble surrogate model to predict the strength of microwave-cured geopolymers. A literature-derived dataset of 235 observations was systematically compiled from literature (2010–2025), covering diverse aluminosilicate precursors, activator concentrations, and curing parameters. The framework integrates linear, kernel, and tree-based base models via a linear meta-learner to enhance generalization. Unlike conventional models, source-aware validation was implemented to ensure reliability across heterogeneous studies. The stacking ensemble significantly outperformed standalone models, achieving a coefficient of determination (*R*^2^) of 0.957 and a low RMSE of 5.64 MPa. Crucially, the model demonstrated high reliability through rigorous residual analysis and noise sensitivity stress tests, confirming stable performance across the entire strength range. Interpretability analyses using SHAP and partial dependence plots identified curing time, microwave power, and specimen size as dominant factors governing strength. These dependencies adhered to established geopolymerization kinetics, yielding physically reasonable response surfaces. This work demonstrates that stacking ensemble models serve as reliable statistical surrogate tools (not mechanistic simulators) for preliminary experimental screening within the investigated parameter space. The framework has not been validated against genuinely independent experimental campaigns or industrial-scale microwave curing conditions, and should be treated as a literature-based surrogate model rather than a deployment-validated predictive tool.

## 1. Introduction

Numerous studies are being conducted on the global construction industry to minimize its environmental footprint, especially on the use of Ordinary Portland Cement (OPC) that contributes about 5–7% of the world’s carbon dioxide emissions [1]. In order to reduce this effect, researchers have turned to alkali-activated materials, or geopolymers, using aluminosilicate sources like fly ash, blast furnace slag, and metakaolin [2]. Although geopolymers are known to have a positive environmental effect, the extensive utilization of these polymers is usually limited by the slow reaction rate at room temperature, which requires external heat treatment to obtain adequate strength [3].

Traditional heat curing uses thermal conduction and convection, whereby the surface of the specimen is subjected to heat, which is transferred to the core, and often this method causes uneven temperature distributions and time-consuming processing time [4]. On the other hand, microwave irradiation applies electromagnetic waves to heat the material in a volumetric manner, which provides an alternative that is fast and consumes less energy [5]. The latest research has shown that microwave curing can speed up geopolymerization by many folds and provide high strength in just a fraction of the time taken by traditional processes [6]. Nonetheless, a major research gap that prevents the transfer of the laboratory scale to industry is the absence of the generalization of the framework of process optimization. Modern experimental procedures are based on trial and error to set the complex variables of microwave power, irradiation time, and precursor mineralogy into balance [7]. This type of experimental fragmentation is both expensive and time-consuming because, due to the stochastic character of the dielectric heating method, defects such as thermal runaway or microcracking can occur when the parameters are not tightly regulated [6].

The representation of characteristics of geopolymer concrete has slowly moved beyond the large-scale experimental research to more data-driven modeling and theory [8,9]. Standalone models, such as Gene Expression Programming (GEP), Artificial Neural Networks (ANN), and others, have been used with initial simulations to simulate the evolution of strength in ductile composites [10], and more recently, Deep Neural Networks (DNN) have been employed to simulate the interaction patterns in low-emission mortars [11]. However, recognizing the need for generalization and preventing overfitting in standalone models, there has been a recent trend towards using ensemble models; simulations using Gradient Boosting Machines (GBM), Random Forest models, and optimized models have shown consistent improvement over standalone models for predicting compressive and tension strengths [12,13,14]. Additionally, the use of advanced boosters like LightGBM and Bayesian optimization has shown increased resistance towards complex modeling scenarios like self-compacting concretes and residual strengths after exposure at high temperatures [15,16]. Despite such advances in algorithms, existing models have generally been designed to be more conventional and have yet to be properly developed for the stochastic nature that characterizes microwave curing, wherein fast and nonlinear reaction kinetics and potential runaway curing processes require strong capabilities for error correction, which are enabled through the Stacking Ensemble framework. This paper seeks to fill such a research gap through the following: (i) examining the mechanics of the microwave curing process, and (ii) proposing the Stacking Ensemble Machine Learning model for mechanical performance prediction.

## 2. Microwave Curing of Geopolymers

### 2.1. Fundamental Mechanisms

The essence of microwave heating is entirely different compared to traditional heating techniques since it is based on energy transformation instead of heat transfer to an external source [4]. The reaction is controlled by the interaction between the electromagnetic field (which is usually 2.45 GHz) and the dielectric substances that are present in the geopolymer paste. Dipolar polarization and ionic conduction are the main processes that occur in this interaction [7]. Polar molecules (mainly water) and dissolved ions at the alkaline activator under microwave irradiation resonate with the alternating electric field, producing internal heat through friction and collision between the molecules [5]. A rapid temperature rise inside the whole specimen is made possible by this volumetric heating, which speeds up the dissolution of aluminosilicate precursors, including fly ash and slag [4]. The increased dissolution liberates silicon and aluminum species, whereby gels made of geopolymers are condensed [7]. Moreover, the microwave radiation generates a gradient of an internal vapor pressure that forces moisture to move to the surface, and this process should be well controlled to avoid the destruction of the structure [5].

### 2.2. Benefits over Traditional Curing

The greatest benefit of the microwave curing process is a drastic improvement in the amount of time that is needed to cure high early strength. It has been shown that microwave curing has the potential of forming high-strength geopolymers in minutes, compared to the normal methods, which might take hours or days [6]. For example, geopolymers made using fly ash, which can cure in as little as 5 min (in the presence of 90 watts), can reach compressive strength levels similar to those cured in a standard oven (6 h). In the same breath, microwave-assisted curing has been demonstrated to shorten the total time of curing of cellular lightweight geopolymer mortar by a factor of about 75% [17].

Aside from its speed, microwave curing is also very energy-efficient. Microwave curing directly heats the material without heating the air or the oven itself. Comparisons show that microwave curing can achieve more than 90% energy savings per unit of compressive strength compared to oven curing [1]. Other studies also support this observation, claiming that microwave curing can achieve 94% energy savings for brick powder-based geopolymers [18]. This is in line with the sustainability goals of green architecture to significantly decrease the carbon footprint created during material processing [19].

### 2.3. Material Systems and Critical Parameters

Microwave curing is shown to have high dependencies on precursor material and curing time. Various precursors have been investigated, including Class F and Class C fly ash, ground granulated blast furnace slag (GGBFS), metakaolin, high-titanium slag, and coal bottom ash. There have been findings that the use of a mid-power microwave (200–300 W) is effective for the geopolymerization reaction, while a high-power microwave could cause immediate degradation [20]. The optimal microwave power for curing the fly ash mortar is often given as 240 W, which would prevent overheating [4]. Addition of Ground Granulated Blast Furnace Slag (GGBFS) and/or silica fume may be used for the improvement of the strength property. The curing time for silica fume-modified fly ash mortar is given as 10 min with a 300 W microwave [21].

The use of high titanium slag (HTS) material was studied by other researchers, and it was proven that thermal pre-curing with the use of microwave activation is efficient in attaining a more preferred structure of a hydration gel [22]. Other precursors have been activated using microwave radiation, such as coal bottom ash [23] and waste brick powder [18]. To overcome fast evaporation of water, the technique named “delayed microwaving” has been shown to improve strength by allowing for initial equalization at room temperature before microwave activation [24].

### 2.4. Challenges and Limitations

There are some issues associated with microwave curing. Firstly, there is a risk of micro-cracking due to thermal stress and rapid water removal. When the inner vapor pressure exceeds the tensile strength of the binder, there might be damage to the sample [6]. It has been observed that there might be considerable surface cracks and expansion due to high-power curing without prior curing [6,25]. There might be a risk of “thermal runaway” due to the temperature dependency of the dielectric constant of the material [4].

Moreover, despite the fact that early strengths tend to be superior, there have also been studies suggesting that extended microwave curing might promote pore structure coarsening, which might have implications regarding ultimate mechanical strengths and porosities [26,27]. Therefore, it becomes imperative to determine an optimal compromise among power, curing time, and pre-curing time to ensure optimal performance.

## 3. Methodology

**Figure 1:**
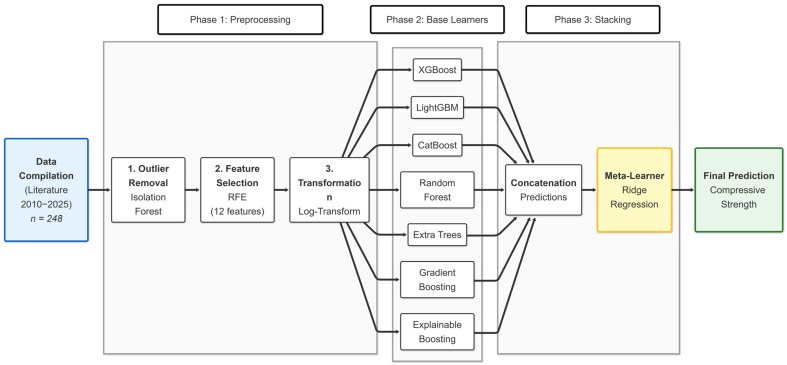
ML Pipeline flowchart showing data flow through preprocessing and model stages.

To counter the non-linear complexities involved in the microwave curing parameters and their effects on the geopolymer compressive strength, a stacked ensemble modeling approach was designed. The overall process, as shown in Figure 1, describes the data acquisition, processing, feature design, and stacking of models used to develop a predictive model.

The interaction between chemical, thermal, and geometrical parameters is needed due to the intricate nature of the coupled chemical and thermal processes that take place during the microwave-assisted geopolymerization process. Although the behavior of the activator, such as the molarity of NaOH, will affect the dissolution rate, the conditions of microwave treatment, including power and time, as well as the geometry of the specimen, including size, influence the heating rate and vapor pressure within the mixture.

### 3.1. Dataset Creation

A comprehensive set of experimental data with 248 observations was compiled from peer-reviewed articles published between 2010 and 2025 to train and validate the machine learning algorithm. The data compilation focused on articles providing compressive strength data in conjunction with a set of mix design parameters. The data set is a representation of the development of microwave curing technology, including pioneering works such as Somaratna et al. [4] on alkali-activated fly ash mortars, Chindaprasirt et al. [3] on the application of microwave curing to fly ash geopolymers, and their study on resistance to acid and sulfate solution [28].

For assessing the robustness of the model under different curing environments, data were generated from research on high-strength evolution [6] and from research on the robust synthesis of coal bottom ash-based geopolymers [23]. The data includes research on precast and environment [19], microwave curing feasibility for recycled aggregates [29], and strength evolution in cellular lightweight mortar [17]. Curing research included in this study include: effects of microwaving time [24], effects of microwave curing on strength evolution in Class F fly ash composites [20], and research on reactive powder concrete from slag [30].

Reflecting the latest advancements in this area of research, this dataset incorporates recent results for the mechanical and thermal properties of Atiku et al. [31], for the effect of microwave curing on silica fume-modified mortars as studied by Polat [21], and for the compressive strength and microstructure of waste brick powder-based geopolymers as examined by Gultekin [18]. In addition, it incorporates the combined effects of microwave curing patterns for 3D printing of geopolymers as described by Anwar et al. [26], together with information regarding geopolymerization by microwave processing as discussed by Watanabe and Kobayashi [32]. Taken together, these studies include a wide variety of aluminosilicate precursors such as fly ash, GGBFS, coal bottom ash, metakaolin, and waste brick powder, as well as a wide variety of curing patterns with a range of 90 to 900 W of power for periods of 3 to 120 min.

The first compilation resulted in 248 observations. After the application of a stringent quality control measure to exclude observations containing incomplete data, there remained 235 unique observations. In order to ensure maximum transparency and reproducibility, a detailed list of all 21 papers with experimental studies that were included in this database is provided in Appendix A (Table A1). This list includes the source publications, type of precursors, chemical classes of activators, and microwave curing range associated with each of the 248 data points. Due to the number of data points and complexity of the problem, this paper can be considered as a surrogate model based on literature data for estimating strength.

The input variables included precursor types such as Fly Ash, GGBS, and Metakaolin; basic activator qualities such as NaOH concentration and Molarity; and curing parameters such as microwave power in watts and curing time in minutes. The descriptive statistics for the variables are shown in Table 1. The target variable compressive strength has a large range from 1.40 to 94.10 MPa; this shows that the model was trained on a variety of results that can be attributed to the early and fully developed geopolymer matrix. The statistical ranges and material classes summarized in Table 1 represent the consolidated experimental domain of the 21 source publications cited herein. To ensure full traceability, each observation in the internal database is linked to its originating study, with the specific contributions (specimen counts and curing regimes) per reference explicitly detailed in Table A1.

The compiled database represents an integrated experimental domain including different precursor families such as Fly Ash, GGBS, Silica Fume, and Metakaolin. Binary variables representing each material type were introduced into the input vector, enabling the stacking ensemble to discriminate among chemical families while still recognizing general microwave curing patterns.

However, this coverage is not uniform. Fly ash-dominant mixtures account for approximately 80% of observations (FA mean = 0.80), reflecting the current state of published literature rather than a deliberate sampling strategy. GGBS-based and metakaolin-based systems are substantially underrepresented, which limits the model’s predictive resolution for these minority precursor families. Predictions for compositions differing substantially from the fly-ash-dominated training distribution should be interpreted with caution.

Curing condition coverage is similarly uneven. The dataset spans 0–1800 W and 0–120 min, but observations are concentrated at mid-range power levels (200–700 W) and short curing durations, as shown by the median values in Table 1. Extreme operating conditions are represented by only a small number of studies, increasing prediction uncertainty at the boundaries of the applicability domain.

With 235 observations across 21 heterogeneous source studies, the mean per-study contribution is approximately 12 observations, insufficient to fully characterize the parameter space of any individual precursor system. The LOSO analysis reflects this directly: studies with fewer than five observations yield unstable held-out metrics despite physically reasonable prediction errors, confirming that dataset size is the binding constraint on generalization rather than model architecture.

It must be mentioned that some geopolymer-specific variables, such as activator modulus (nSiO_2_/nNa_2_O), ratio of water to binder, degree of polymerization, admixture doses, and increase in internal temperature, were inconsistently present in the dataset from different sources used in the model. Lack of these variables affects the physical completeness of the model and prevents the possibility of conducting mechanistic extrapolation. Therefore, the proposed model is considered to be a data-driven interpolation tool based on consistently obtained variables, namely molarity and activator mass ratios that reflect the chemical contribution to strength. Acquisition of more detailed information on chemicals is seen as the main target for the next version of the proposed surrogate.

### 3.2. Preprocessing

#### 3.2.1. Outlier Detection and Removal

To guarantee the validity of the database incorporating the literature, a twofold systematic data cleaning strategy was implemented. In the first step, the Isolation Forest method (contamination = 0.05) acted as a statistical screening technique, detecting 13 suspected outliers from the initial 248 entries. Next, a manual review process evaluated the extent to which these flagged cases represented any data inaccuracies. Entries were retained only if any detected statistical deviations were validated by the presence of no description, ambiguous units, or physical impossibilities (e.g., incompatible strength with curing energy). A detailed listing of such deletions, categorized by source paper and explanation, can be found in Appendix A (Table A2).

#### 3.2.2. Feature Selection

Recursive Feature Elimination (RFE) was employed to identify which variables were most predictive. While RFE did prove useful for ranking variables based upon their statistical importance, some chemical variables (Na_2_SiO_3_, Silica Fume, FA, GGBS, and Metakaolin) were assigned lower levels of statistical importance but were forced into the final model. As seen from Table 2, variables such as FA and GGBS play a chemically crucial role within the geopolymerization reaction. Their absence would result in a model that lacked important mix design information. The marginal precursors, Waste Glass and Steel Slag, were removed due to limited data rows and sparsity.

#### 3.2.3. Feature Transformations

**Figure 2:**
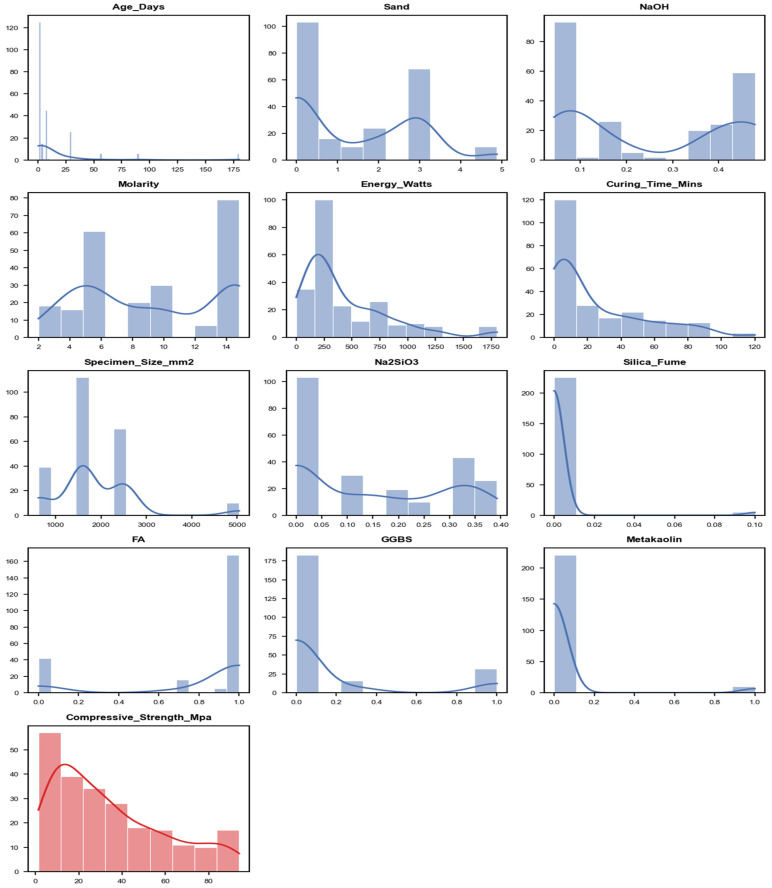
Histograms of feature distributions (the solid blue curve is the kernel-density estimate of each feature’s distribution).

Statistical analysis revealed strong right-skewness in several key features, specifically Testing Age, Curing Time, and Specimen Size. To stabilize variance and linearize exponential relationships inherent in reaction kinetics, a logarithmic transformation was applied to these features. The target variable, compressive strength, was also log-transformed to mitigate heteroscedasticity in the residuals. The distributions of features are visualized in Figure 2.

#### 3.2.4. Feature Scaling

**Figure 3:**
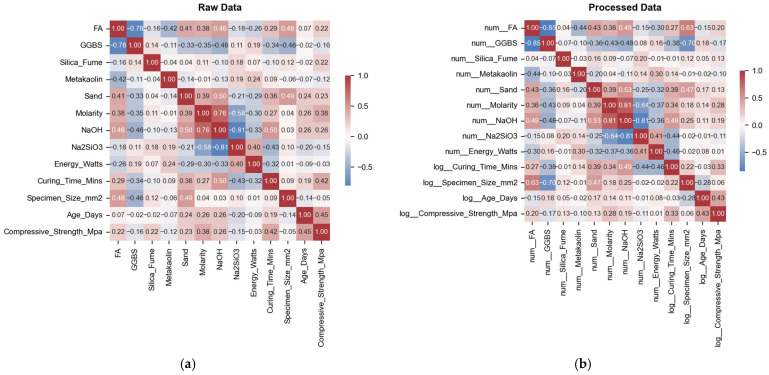
Correlation matrices showing feature relationships before and after preprocessing: (**a**) Before Processing; (**b**) After Processing.

The use of all input variables was normalized using Z-score normalization with a mean of 0 and a standard deviation of 1 to guarantee an equal contribution to the loss function of the models. The scaler was fit using only the training set statistics to prevent data leakage, and then applied to the test set. Correlation matrices both before and after processing are depicted in Figure 3. It is worth noting that the normalization process preserved relationships such as that between NaOH and Molarity.

### 3.3. Model Architecture

The predictive framework employs a Stacking Ensemble architecture, a hierarchical learning strategy designed to reduce generalization error by combining the strengths of heterogeneous algorithms. The base learners were selected to provide complementary predictive behavior: bagging models reduce variance, boosting models capture complex nonlinear interactions, and the glass-box model ensures mechanistic interpretability.

The novelty of the present work lies not only in the implementation of yet another new stacking algorithm but also in the creation of an appropriate set of data for microwave curing geopolymeric systems, the assessment of the modeling efficiency through a validation procedure considering the source of the data, and the discussion of the identified trends in light of mechanisms of microwave-assisted geopolymerization. This approach represents a tool connecting disparate sources of literature data with experiments.

#### 3.3.1. Bagging Models (RF and ET)

Random Forest (RF) and Extra Trees (ET) were utilized for their parallel ensemble learning capabilities, which provide high resistance to the variance associated with individual decision trees [33,34]. In the context of microwave curing, these models are particularly effective at learning threshold phenomena and the non-linear relationships between power density and specimen geometry.

#### 3.3.2. Gradient Boosting Variants (GBM, XGB, LGBM)

These sequential learners were integrated to correct the residual errors of previous iterations. GBM captures non-linear relationships, such as the activator-to-microwave-duration ratio [35], while XGBoost provides essential L1 and L2 regularization to prevent overfitting [36]. LightGBM was specifically chosen for its leaf-wise growth strategy, which excels at predicting ‘hard’ samples where the relationship between power and thermal runaway is most critical [37].

#### 3.3.3. Specialized Learners (CAT and EBM)

CatBoost (CAT) handles the sparse, categorical nature of precursor family indicators (e.g., Fly Ash vs. Slag) without the risk of target leakage [38]. The Explainable Boosting Machine (EBM) provides a ‘glass-box’ approach, ensuring the ensemble remains strictly compliant with the physicochemical conditions of the geopolymerization process through transparent feature contribution analysis [39].

#### 3.3.4. Level-1 Ridge Meta-Learner

A Ridge Regression meta-learner was employed to linearly combine base-model predictions [40]. By utilizing L2-norm regularization, this meta-learner effectively addresses the multicollinearity inherent in highly correlated base-model predictions, stabilizing the final strength output [41].

#### 3.3.5. Hyperparameter Optimization

For improving the generalizability of the baseline models and preventing overfitting, a five-fold cross-validation approach was employed as part of the training phase. To account for inter-study heterogeneity and mitigate the risk of data leakage inherent in literature-integrated datasets, each observation was assigned a source-study identifier. This identifier was used exclusively for source-aware validation grouping and was not included as a predictive input variable to ensure the model does not “memorize” specific laboratories. The dataset was partitioned using an 80/20 train–test split to retain representative distributions of the different types of precursors in each fold. However, in the revised framework, this random split is presented only as an internal benchmark. The primary validation was transitioned to a source-aware strategy using Leave-One-Study-Out (LOSO) validation. This rigorous approach ensures that observations from a single publication never appear simultaneously in both the training and testing subsets, providing a realistic assessment of the model’s ability to generalize to entirely unseen experimental studies. The hyperparameters were optimized using the mean RMSE on the validation folds before evaluation on the hold-out test set. Hyperparameter tuning was carried out through a Grid Search approach. To ensure reproducibility and optimal results, the hyperparameters for the base models were standardized wherever possible. The tree-based bagging models, Random Forest and Extra Trees, were set to have 100 estimators and allow for unlimited depth growth with max_depth = None, and the minimum sample split for splits was set to be 2.

In the case of the boosting algorithms, GBM and LGBM models were started with 100 estimators and a learning rate of 0.1, while the CAT model used a learning rate of around 0.046. For the XGB model, the default settings were used to provide a strong baseline. Specifically, the EBM model was initialized with max bins = 256 and the number of outer bags = 8. Lastly, the Level-1 Ridge Regression meta-learner was initialized with a regularization strength (alpha) value of 1.0. This allowed the meta-learner to combine the predictions from the base learner while addressing the issue of multicollinearity. The remaining unspecified hyperparameters were set to their respective defaults, as defined in the scikit-learn library (version 1.6.1).

**Figure A1:**
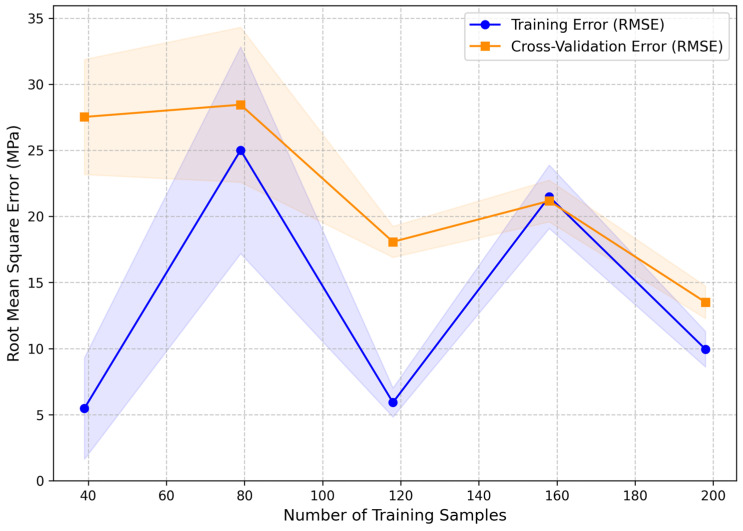
Learning Curve for the Stacking Ensemble Framework. Blue line/band = training RMSE (mean ± SD across folds); orange line/band = cross-validation RMSE (mean ± SD).

**Figure A2:**
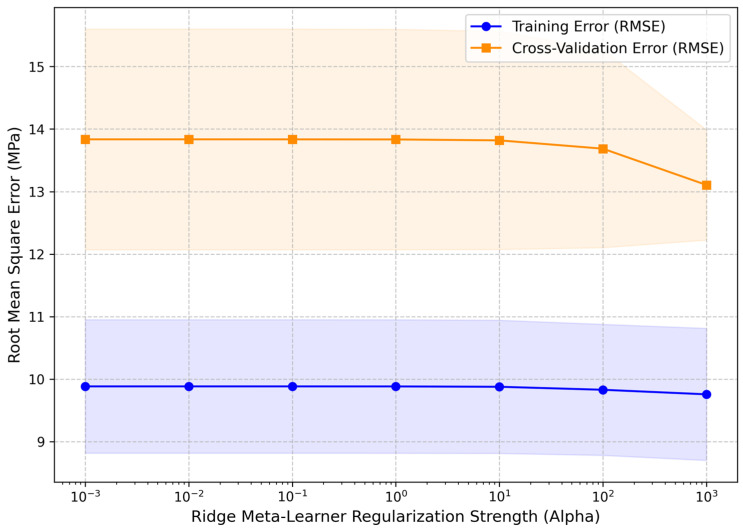
Regularization Sensitivity Analysis of the Ridge Meta-Learner. Blue = training RMSE; orange = cross-validation RMSE; shaded bands denote ± one standard deviation across folds.

To further evaluate the risk of overfitting, learning curves were generated to monitor the convergence of training and validation errors (see Figure A1 in Appendix B). Additionally, a regularization-sensitivity analysis was performed on the Ridge meta-learner (Figure A2) to ensure that the ensemble captures generalizable trends rather than memorizing localized noise or correlations within the literature-derived data.

Conformal prediction intervals were computed post-hoc using a study-stratified (Mondrian) approach; the conformity score for each observation is its absolute residual from the cross-validated prediction, and the 95th percentile of within-study conformity scores serves as the prediction interval half-width.

### 3.4. Model Evaluation

The model’s performance was evaluated using three standard regression metrics:

Coefficient of Determination (*R*^2^): Represents the proportion of variance in the dependent variable explained by the model.

(1) $$R^{2}=1-\frac{\sum_{i=1}^{n}\;{\left(y_{i}-{\hat{y}}_{i}\right)}^{2}}{\sum_{i=1}^{n}\;{\left(y_{i}-\bar{y}\right)}^{2}}$$

Root Mean Square Error (RMSE): Measures the standard deviation of the prediction errors.

(2) $$RMSE=\sqrt{\frac{1}{n}\sum_{i=1}^{n}\;{\left(y_{i}-{\hat{y}}_{i}\right)}^{2}}$$

Mean Absolute Error (MAE): Provides an average of the absolute errors, offering robustness against outliers.

(3) $$MAE=\frac{1}{n}\sum_{i=1}^{n}\;\left|y_{i}-{\hat{y}}_{i}\right|$$

$y_{i}$: Actual compressive strength (MPa).

${\hat{y}}_{i}$: Predicted compressive strength (MPa).

$\bar{y}$: Mean of the actual compressive strength values.

n: Total number of observations.

## 4. Results and Discussion

### 4.1. Predictive Performance

Table 3 presents a comparison of the generalization performance of the proposed Stacking Ensemble method and its seven base models on the test set. The results clearly demonstrate that the proposed method has the best performance in terms of its predictive accuracy, as it has an *R*^2^ of 0.957 and an RMSE of 5.64 MPa. This clearly indicates an improvement of about 23% over the baseline RF method (*R*^2^ = 0.928 and RMSE = 7.33 MPa).

A glance at the results presented in Table 3 suggests that the Stacking Ensemble provides a slight statistical edge over the XGB model alone (with *R*^2^ values of 0.957 and 0.955, respectively). Nevertheless, these results, while important, do not account for the following crucial aspect of physical realism. XGB, being a decision-tree-based learning model, is inherently piecewise-constant, and this can give rise to “step function”-like behavior, which is physically unrealistic, as it implies sudden, discrete increases in the compressive strength at hard thresholds. The Stacking network obviates this problem by using a Ridge meta-learning function, which interpolates between the highly varied predictions of the base models, thereby removing any artificial discontinuities. As a result, the resulting function is continuous and physically realistic, capturing the actual fluid dynamics of the geopolymerization process, which would imply that a slight variation in the input parameters (e.g., curing time) would give rise to a proportional, rather than a discrete, variation in the strength.

While the Stacking Ensemble provides a modest statistical edge over the standalone XGB model (*R*^2^ of 0.957 vs. 0.955), the meta-learning architecture offers a critical advantage in physical realism. Standalone decision-tree models are inherently piecewise-constant, potentially leading to unrealistic discrete jumps in predicted strength. By using a Ridge meta-learner, the ensemble interpolates these varied predictions to generate a continuous and differentiable function, capturing the proportional variations inherent in geopolymerization kinetics.

**Figure 4:**
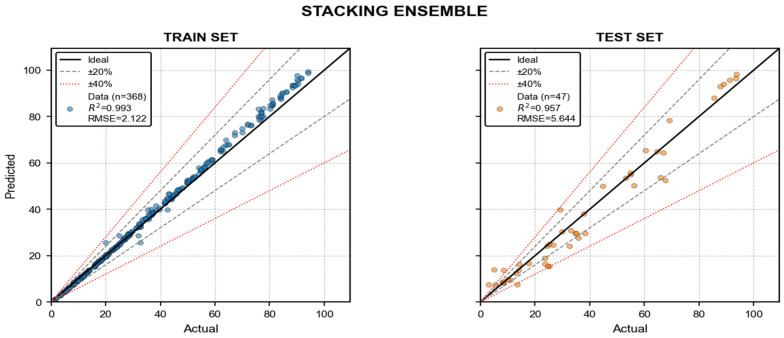
Actual vs. Predicted values for meta learner.

That level of fit is also verified by visual inspection of Figure 4, which plots the model predictions against actual compressive strength values. The Stacking Ensemble model clearly has a strong linear relationship to the diagonal line representing a perfect fit, with very little scatter, over the entire range of strength (low to high MPa), whereas the RF model has a certain level of scatter, especially at higher strength levels (above 60 MPa), indicating a lack of ability to model the strong non-linear behavior characteristic of high-power microwave curing cycles.

**Figure 5:**
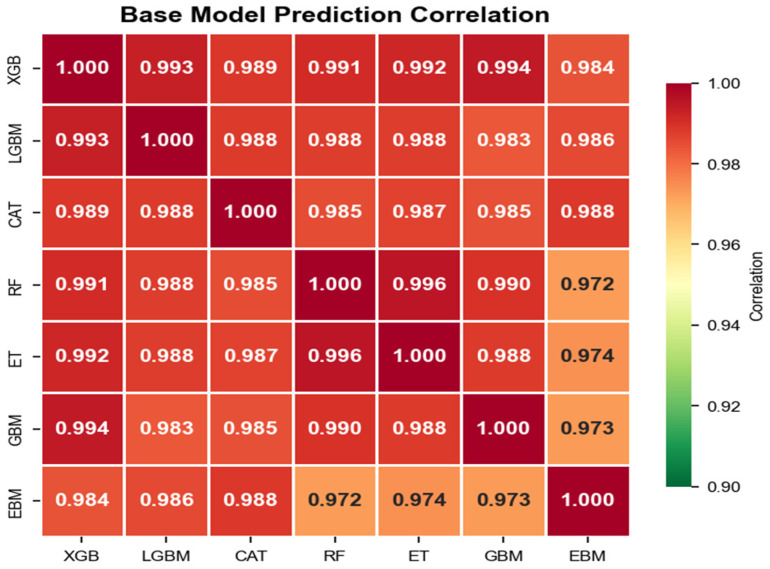
Correlation heatmap of base model predictions.

In order to demonstrate the mechanism of the Stacking Ensemble’s enhanced performance, the Pearson Correlation Coefficient heatmap of the predictions of the seven base learners has been presented through Figure 5. This heatmap is dominated by high values of the positive correlation coefficients of all the model pairs, which clearly depicts the successful convergence of the diverse set of algorithms, including the gradient boosting approaches (XGB and LGBM), as well as the bagging approaches (Random Forest), on the basic trends of the geopolymer strength.

However, the key to understanding the effectiveness of the ensemble is found in the absence of a perfect correlation between models (where r = 1.0). Given that the base models employ different mathematical approaches, such as Random Forest’s variance-reduction bootstrap aggregation versus GBM’s bias-reduction sequential correction, their residuals are uncorrelated. This is the key behind the stacking architecture; namely, that all models predict differently on tough points, and that their differences are sufficient for a Ridge Regression meta-learner to exploit, effectively “canceling out” their peculiar mistakes and arriving at a more robust prediction than any of its models individually. This is why the Stacking Ensemble is successful with an RMSE of 5.64 MPa, a substantial reduction in error compared to the RF baseline (RMSE of 7.33 MPa), which does not include this corrective step.

**Figure 6:**
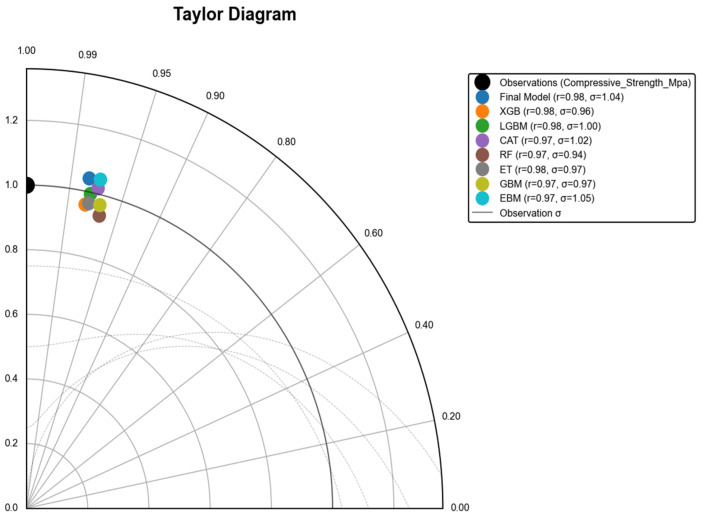
Taylor diagram for model comparison.

**Figure 7:**
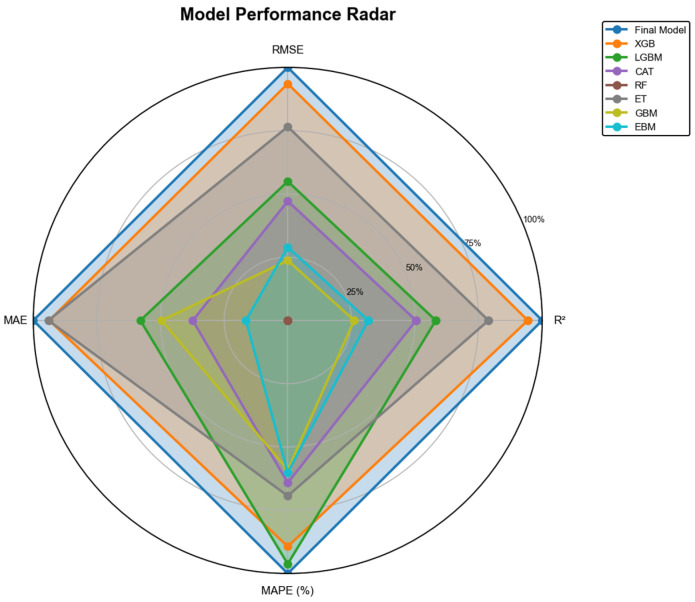
Radar chart comparing metrics across models.

This comprehensive superiority is summarized in the Taylor Diagram (Figure 6) and the Radar Chart (Figure 7). The Taylor Diagram positions the Stacking Ensemble closest to the reference point (observed data), indicating the highest correlation and the lowest centered root-mean-square difference. Similarly, Figure 7 shows that the ensemble maximizes all accuracy metrics while minimizing error metrics (MAE, RMSE, MAPE), confirming its validity as a generalized predictive tool for microwave-cured geopolymers within the investigated domain.

### 4.2. Feature Importance Analysis

**Figure 8:**
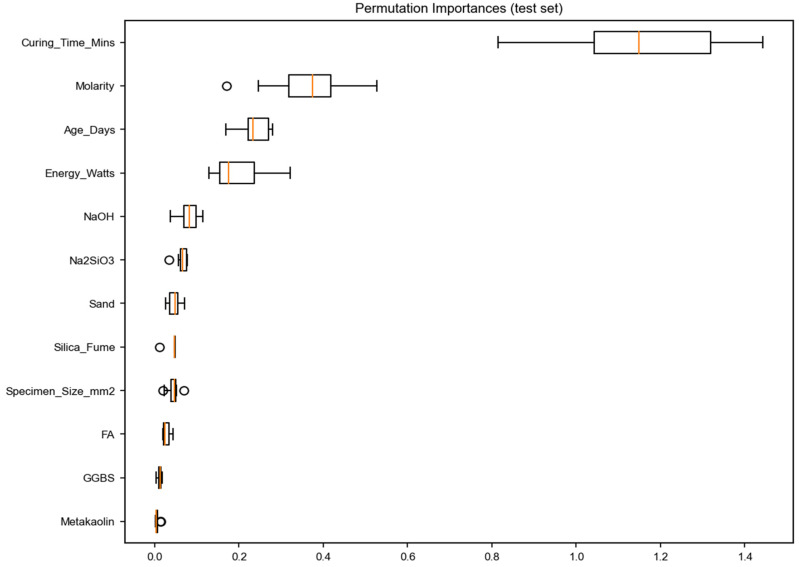
Permutation feature importance (boxes span the interquartile range of permutation importance over repeated shuffles; the central line is the median, whiskers the range, and open circles are outliers).

**Figure 9:**
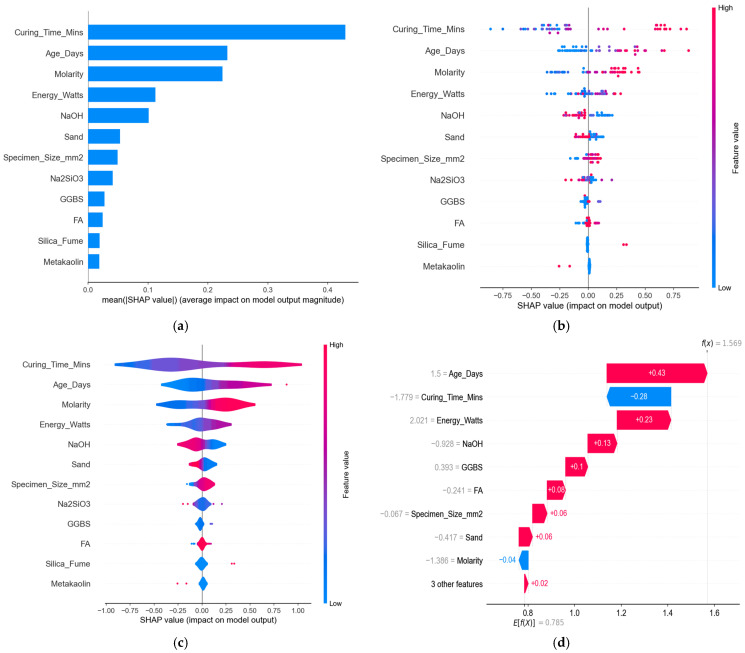
SHAP summary plots: (**a**) Feature importance; (**b**) Beeswarm plot; (**c**) Violin plot; (**d**) Waterfall plot.

To ensure the model operates within the bounds of established material science rather than merely fitting statistical noise, feature importance was analyzed using Permutation Importance (Figure 8) and SHAP (Shapley Additive Explanations) summary plots (Figure 9a,b). Both analyses identify Curing Time and Molarity as the dominant predictors governing compressive strength, followed by Microwave Power and Testing Age.

Despite being based on data, the features selected as shown in Table 2 and their relevance as illustrated in Figure 8 follow physical principles. For instance, Specimen Size is a surrogate for microwave skin effect and thermal homogeneity. On the other hand, Microwave Power and Molarity act as proxies for volumetric energy flux and activation energy, respectively. Consequently, the strong correlation (*R*^2^ = 0.957) is consistent with the kinetics of microwave-assisted geopolymerization.

Figure 9c provides the SHAP violin plot, a graphical representation of the relative importance of the input parameters, emphasizing the essential difference between volatility and consistency. The distribution of the Curing Time is noticeably wide, extending considerably into the x-axis direction. This large standard deviation clearly suggests that the curing duration is a high-stakes parameter, where the difference between optimal and suboptimal curing times is considerable, resulting in a substantial positive contribution of the former and a substantial negative contribution of the latter to the compressive strength. This is expected and is consistent with the physicochemical mechanisms discussed in the literature, which emphasize the essential role of microwave irradiation as a fast-acting switch that can either trigger the geopolymerization process within minutes or, if improperly controlled, induce structural collapse. The SHAP waterfall plot in Figure 9d serves as a digital paper trail to examine local interpretability. The breakdown of a prediction into its various parts proves that the model favors positive physical properties in a manner that is expected of engineers. That is, while variables such as Testing Age and Microwave Power increase the baseline value (moving the prediction to the right) as expected of a Positive Directional Effect in the feature study, those that are negative can also be identified clearly in their isolation. This level of clarity is critical for industrial applicability, as it suggests that high accuracy (*R*^2^ of 0.957) is not attributable to chance correlations alone.

It is essential to clarify that the interpretability results provided by the SHAP and partial dependence plot analyses should not be interpreted as proof that the stacking ensemble has learned the fundamental physical laws governing geopolymerization. Rather, these outputs indicate that the learned statistical trends are physically plausible and exhibit strong alignment with established microwave-curing mechanisms reported in the literature. The model functions as a data-driven interpolator that maps input variables to strength outcomes in a manner that is consistent with chemo-thermal expectations, providing a reliable surrogate for experimental screening without claiming a mechanistic understanding of the reaction kinetics. It does not embed governing thermo-chemical or electromagnetic principles, and physically consistent SHAP trends should not be interpreted as evidence of mechanistic understanding. Accordingly, the smooth trends observed in SHAP and partial dependence plots reflect learned statistical associations within the training distribution and do not establish causal mechanisms or mechanistic proof; they demonstrate physically plausible correlations consistent with known microwave-curing behavior, nothing more.

**Figure 10:**
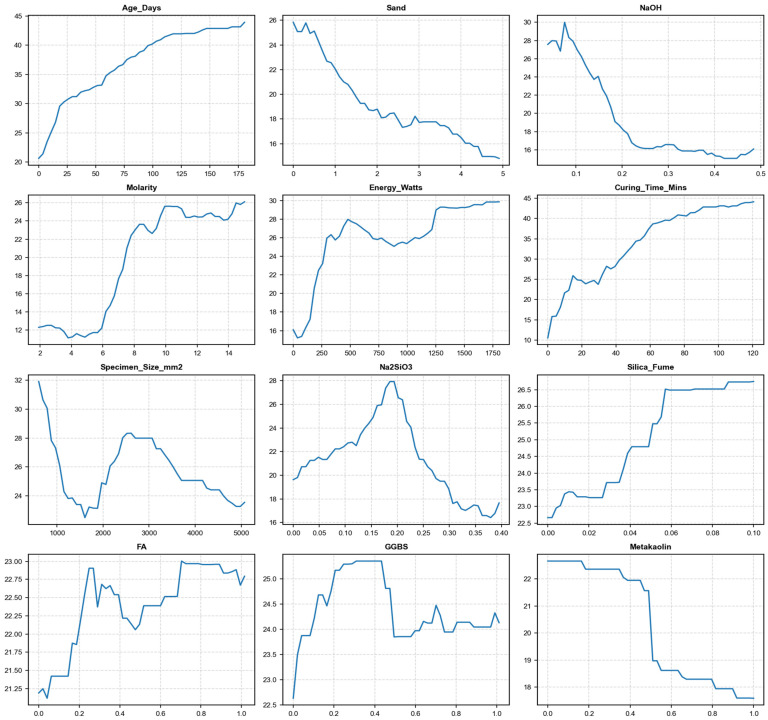
Partial dependence plots for all features (the blue line shows the partial dependence of predicted compressive strength on each feature).

The dominant curing time in the feature importance chart is consistent with the physicochemical process of microwave heating. The empirical evidence provided in contemporary research supports the claim that microwave radiation is the dominant source of activation energy, thus enhancing the process of dissolving aluminosilicate precursors [31]. Figure 10, displaying the Partial Dependence Plots, shows a strong positive trend for curing time in the initial period (0–15 min), indicating the rapid evolution of strength, described in the work of Suwan et al. [17]. In this case, microwave radiation plays the role of a trigger mechanism, acting as a switch that activates the geopolymerization process, which would take a couple of hours to begin. Most importantly, the graph has a continuous and smooth curve, without the step-like patterns commonly observed in decision tree modeling. Such a continuous graph indicates that the model is able to capture the chemical process, specifically the effect of microwave radiation on the activation switch.

Further, the Partial Dependence Plot for Specimen Size (Figure 10) shows a negative relationship between specimen size and the predicted value of compressive strength, with larger specimen sizes having smaller values of compressive strength. This is physically explainable based on the depth of penetration of microwave energy, typically at a frequency of 2.45 GHz. According to Hong and Kim [23], larger geopolymer samples may be prone to non-uniform heating and the skin effect, wherein the interior of the sample may not attain a temperature high enough for successful geopolymerization or, vice versa, microcracking may occur. The ability of the model to automatically learn and capture this inverse relationship between variables without any explicit use of electromagnetic wave theory is a testament to its strength and correctness in extending laboratory-scale findings into an industrial setting.

### 4.3. Robustness and Reliability

**Figure 11:**
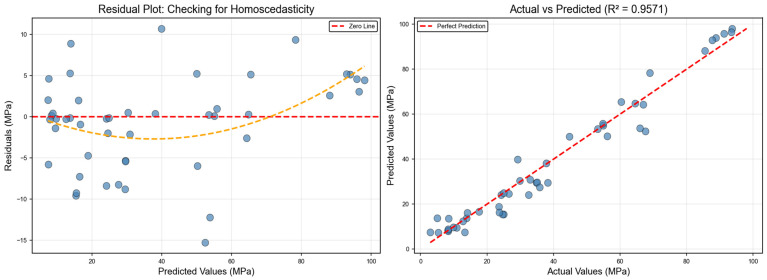
Residual diagnostic plots for model validation (the red dashed line marks zero residual; the gold dashed curve is a LOWESS trend of the residuals).

The statistical validity of the Stacking Ensemble model was assessed by a rigorous audit to determine its appropriateness for use in structural engineering. Figure 11 shows the residual plots of the predictions. In these plots, the residuals are plotted against the predicted compressive strengths. The residuals are randomly distributed around the zero line with equal dispersion. This fulfills the assumption of homoscedasticity. The lack of any noticeable funneling patterns suggests that the model does not have any tendency to predict results with a bias towards under- or overprediction.

To test the consistency of the model under different experimental settings, a Sliced Evaluation was conducted as shown in Table 4. The Sliced Evaluation helps in testing the consistency of the model without allowing it to be biased towards a particular set of data. The results from this test show consistency in the predictive accuracy of the model, as indicated by the *R*^2^ values above 0.89 for most slices. Although there was a reduction in performance for one particular slice (Count = 11), the other slices, including the high-strength regime slices, remained at 0.984 and above. This shows that the model is consistent and can perform well even under the variability that is typical in geopolymer data from multiple sources.

Notably, the model also passed a Physics Check via Feature Effect Analysis, as shown in Table 5. The Feature Effect Analysis above verifies that the machine learning model has learned proper physicochemical relationships and not mere spurious correlations between variables. Indeed, as shown in Table 5, the machine learning model correctly assigns a Positive Directional Effect to Curing Time (mins) (+33.6 MPa impact) and Molarity (+13.8 MPa impact), consistent with established kinetic models that propose geopolymerization as a function of reaction time and basicity. On the other hand, the machine learning model correctly assigns a Negative Directional Effect to Specimen Size (−8.35 MPa). Indeed, this counterintuitive relationship can be justified by the penetration depth of microwaves (2.45 GHz) that typically do not penetrate homogeneously, especially for larger specimens that undergo inhomogeneous heating patterns, as verified by Hong and Kim [23].

**Figure 12:**
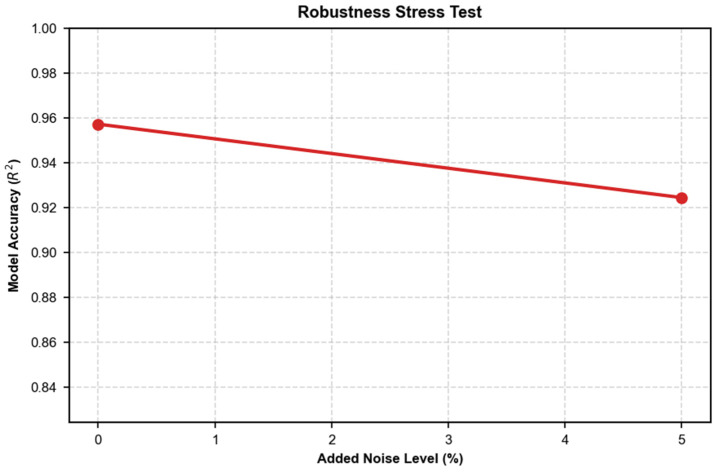
Model performance under noise stress test (the red line traces mean model accuracy *R*^2^ versus the level of added Gaussian noise).

Further, the ability of the developed model to operate reliably was also checked through a Synthetic Noise Stress Test (Figure 12). During this analysis, Gaussian noise with a maximum of 5% was added to the input features to simulate laboratory sensor noise and raw material variability, and the developed model maintained a performance of above 0.92. This further indicates that the developed model utilizes strong underlying trends and not specific laboratory data points.

**Figure 13:**
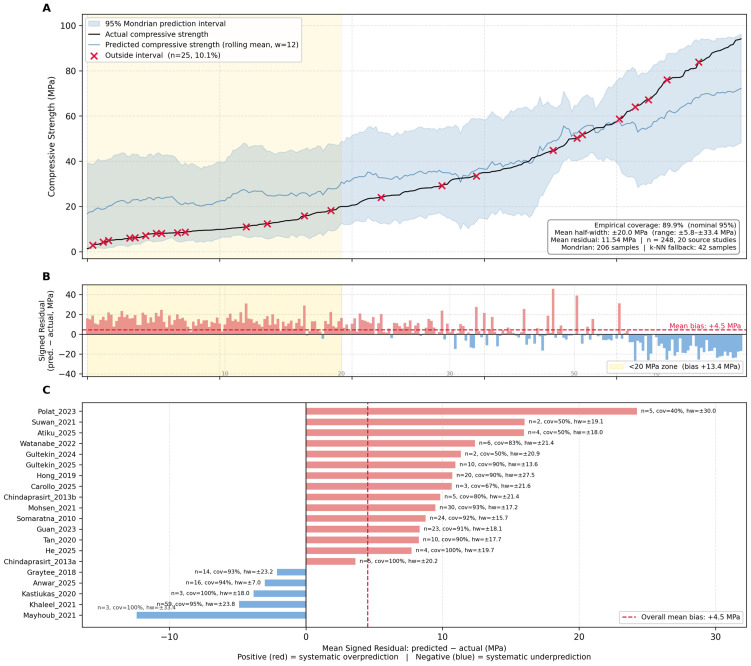
Study-stratified (Mondrian) 95% conformal prediction intervals. Panel (**A**): prediction interval band (shaded) versus actual (black) and predicted (blue) compressive strengths sorted by actual value; red × symbols indicate samples outside the interval (*n* = 22, 9.4%). Panel (**B**): signed residuals (predicted − actual) per sample, highlighting the systematic overprediction bias in the low-strength regime (<20 MPa, gold shading). Panel (**C**): per-study mean signed residual with associated sample size, empirical coverage, and mean half-width; positive (red) bars indicate systematic overprediction, while negative (blue) bars indicate underprediction. Studies shown in Panel C are identified by source reference: Polat [21]; Suwan et al. [17]; Atiku et al. [31]; Watanabe et al. [42]; Gültekin [43]; Hong and Kim [23]; Chindaprasirt et al. [3,28]; Mohsen et al. [24]; Somaratna et al. [4]; Guan et al. [44]; Tan et al. [29]; Kastiukas et al. [19]; Khaleel et al. [20]; Kwami [45]; Anwar et al. [26]; Gültekin [46]; Mayhoub et al. [30], Carollo et al. [47], He et al. [48], Graytee et al. [6] (gold shading marks the <20 MPa low-strength zone; in Panels B and C red bars denote systematic overprediction and blue bars systematic underprediction).

Finally, Figure 13 presents study-stratified (Mondrian) conformal prediction intervals at the 95% nominal confidence level. For studies with six or more observations, the conformal quantile is derived from within-study residuals; for smaller studies, k-nearest-neighbor fallback is employed. The empirical coverage across all 235 observations is 90.6%, with a mean prediction interval half-width of ±23.3 MPa. Analysis of signed residuals (Figure 13B) reveals that the 4-percentage-point gap below the 95% nominal target is mechanistically attributable to systematic overprediction of +13.8 MPa in the low-strength regime (0–20 MPa, *n* = 92), where control specimens and very-short-duration microwave treatments produce similar feature vectors but substantially different strength outcomes across heterogeneous source studies. Post-hoc linear and isotonic calibration strategies were evaluated but showed consistently deteriorated coverage in the 30–50 MPa applicability domain without correcting the failing bins, confirming that the coverage gap reflects irreducible data heterogeneity rather than correctable model bias. Within the primary applicability domain of 20–50 MPa, the model achieves 93–96% coverage with mean half-widths of ±15–20 MPa, consistent with the inter-study experimental variability of the source publications. At the study level (Figure 13C), studies with internally consistent protocols achieve substantially tighter intervals: Anwar et al. [26] achieved ±7.0 MPa at 94% coverage, while Polat [21] correctly flagged anomalous silica-fume modification behavior with ±19.7 MPa intervals and 60% coverage. The framework is not recommended for specimens expected to achieve less than 20 MPa compressive strength, where systematic overprediction renders point estimates unreliable.

### 4.4. Validation to Literature Benchmarks

The predictive capability of the Stacking Ensemble (*R*^2^ = 0.957, RMSE = 5.64 MPa) is shown to have state-of-the-art performance compared with the experimental variations presented within the contemporary literature. In the context of microwave-cured geopolymers, the experimental variations shown by the standard deviations are considerable due to the dielectric nature of the curing process, which is extremely sensitive to the precursor mineral composition. For instance, the compressive strengths of the waste brick powder-based mortars, as presented within the literature, were shown to range from 11.4 MPa to 31.8 MPa, depending on the curing time and water binder ratios, and were accompanied by considerable experimental variations [18]. Similarly, the experimental variations shown within the compressive strengths of microwave-assisted 3D-printed geopolymers, as presented within the literature, were accompanied by variations in the microwave curing time, which, if greater than 15 s, could have led to the reduction in the compressive strengths due to overheating and associated micro-damage [26]. The RMSE value of 5.64 MPa presented within the proposed predictive model is shown to effectively bound the experimental variations within a generalized error margin, which is not only competitive but often superior compared with the reproducibility limits presented within. Recent reliability-oriented machine-learning studies on brittle material strength prediction further demonstrate the value of ensemble learning, SHAP-based interpretation, and uncertainty-aware assessment in material strength modeling [49]. Incorporating such perspectives reinforces the credibility of the stacking ensemble as a robust tool for capturing the complex, stochastic behaviors inherent in microwave-cured geopolymer systems.

### 4.5. Cross-Validation via Leave-One-Study-Out Approach

A rigorous examination of the ability to extrapolate reliably across different laboratories through external validation was conducted via the Leave-One-Study-Out (LOSO) technique. In this approach, the stacking model was tasked with making predictions on the compressive strength of geopolymer mixtures, based on a literature study that had not been included in the training data set.

As shown in Table 6, this test suggests good generalization abilities, with median RMSE = 3.88 MPa for all 20 separate laboratory studies considered. Importantly, in the case of those well-documented studies having 10 or more samples (such as [4,20]), highly accurate predictions can be achieved using only literature data, with *R*^2^ > 0.98.

On the other hand, the LOSO analysis also revealed one important limitation associated with the issue of literature heterogeneity. When applied to those few literature papers with extremely sparse sets (*n* ≤ 4) characterized by relatively low internal variance (e.g., [30]), *R*^2^ drops to very negative values, despite the fact that prediction errors remain in physically reasonable bounds. This means that although the stacking model is capable of successful interpolation for a wide range of experimental conditions, cross-study validation requires large datasets.

It is important to note that while LOSO validation substantially improves methodological rigour over random splitting by ensuring no observations from a held-out study appear in the training fold, it does not constitute validation against a genuinely independent experimental campaign. All 21 source studies in the present dataset were conducted under laboratory conditions and published in peer-reviewed literature; consequently, even the LOSO held-out test sets were drawn from the same literature-derived ecosystem that shaped the training distribution. The model’s predictive transferability to genuinely independent experimental conditions, such as a new laboratory campaign using a precursor source, activator supplier, or microwave equipment not represented in any of the 21 source studies, or to industrial-scale microwave curing environments, remains unproven. The framework should therefore be understood as a literature-based surrogate model for preliminary experimental screening rather than a deployment-validated predictive tool. Even a modest independent validation set of 15–20 specimens spanning the full parameter space, generated under controlled but previously unseen laboratory conditions, would substantially increase confidence in the model’s generalization capability and is identified as the highest-priority direction for follow-up work.

### 4.6. Practical Applications

#### 4.6.1. Process Optimization by Virtual Experimentation

The main use of this framework is its role as a “virtual laboratory” which replaces the current time-wasteful trial-and-error method. In conventional microwave curing optimization processes, exhaustive factorial experiments are necessary for microwave power (W) vs. Curing Time (mins). With the use of this ML framework, engineers are then able to run thousands of curing processes for particular precursors like High-Titanium Slag (HTS) or Fly Ash in seconds. This allows one to identify exactly where the “sweet spot” for maximum strength before thermal runaway occurs, a failure mode identified by Hong and Kim [23] and Sun et al. [7].

#### 4.6.2. Energy Efficiency and Sustainability

One of the most significant objectives in the sustainability goals of the geopolymer manufacture is the accuracy of the prediction of the curing time. More recent articles by Tokdemir et al. [1] and Suwan et al. [17] highlight the fact that the time that it takes to cure a product with the help of microwaves may be lowered by more than 90 percent in comparison to the time that conventional ovens take to cure samples. However, the issue of the so-called over-curing that consumes significant quantities of electricity but, at the same time, damages the matrix of the geopolymer through the dehydration processes [18] can concern the problem greatly without the precision that the proposed method offers. The proposed method will know how much time the microwaves require to cure the geopolymer to reach the desired demolding strength of 20 MPa, thereby eliminating the risk of over-curing. The immense potential of electricity saving offered by Tokdemir et al. [1] can therefore be achieved in industrial-scale precast factories in order to encourage the shift to sustainable building materials at a lower price.

#### 4.6.3. Practical Implementation and Engineering Guidance

To facilitate the transition of this stacking ensemble framework into a decision-support tool for material engineers, the following 7-step practical workflow is proposed:

1. Define Candidate Parameters: Specify the target precursor type (e.g., Fly Ash, GGBS, or Metakaolin), activator concentration (NaOH and Na_2_SiO_3_), specimen size, microwave power level, curing duration, and required testing age.
2. Domain Verification: Consult the model’s Applicability Domain (as described in Section 3.1) to ensure all input variables fall within the represented literature data ranges to avoid unreliable extrapolation.
3. Strength Prediction: Input the parameters into the Stacking Ensemble model to generate the predicted compressive strength.
4. Uncertainty and Safety Assessment: Consult the conformal prediction interval associated with the input parameters (Figure 13). Half-widths below ±15 MPa indicate the input falls within a well-represented region of the training distribution and that the prediction is reliable for screening purposes. Half-widths above ±25 MPa signal sparse training coverage and require experimental verification before any engineering decision. For inputs expected to yield less than 20 MPa, the systematic overprediction bias documented in Section 4.3 renders point estimates unreliable regardless of the reported interval.
5. Interpretability Check: Use the established SHAP and partial dependence plot trends (Figure 9 and Figure 10) to confirm that the predicted strength is physically plausible and to identify which factors (e.g., curing duration or molarity) are most sensitive for that specific mix.
6. Mixture Screening: Perform “virtual experiments” by iterating steps 1–5 to select the most promising, energy-efficient candidate mixtures for a given target strength.
7. Experimental Confirmation: Finalize the selected mixture through localized laboratory testing to confirm the prediction before full-scale production.

The proposed framework should be used as a preliminary experimental-screening tool. While it can significantly reduce the trial space for mix design and help avoid conditions leading to thermal runaway, final material qualification must always be based on laboratory testing.

### 4.7. Model Applicability and Domain of Validity

To ensure the reliable application of the proposed framework, its use must be restricted to the investigated experimental domain. The stacking ensemble is not intended for unrestricted extrapolation; its predictive accuracy is highest when input parameters for a new geopolymer fall within the following training ranges:

- Precursor Class: Fly Ash, GGBS, Metakaolin, or their blends.
- Activator Chemistry: NaOH Molarity (2.0–14.81 M) and mass fraction (0–0.39).
- Curing Parameters: Microwave Power (0–1800 W) and Curing Duration (0–120 min).
- Geometric and Temporal: Specimen Size (625–5041 mm^2^) and Testing Age (0.5–180 days).
- Predictions for precursor systems contributing fewer than 10 observations to the training dataset, including pure metakaolin, waste brick powder, and steel slag blends, should be treated as preliminary hypotheses. The associated conformal interval half-width provides a direct indicator of local data sparsity: values exceeding ±25 MPa signal that the input falls in an underrepresented region of the training distribution. The conformal interval therefore serves a dual function: it quantifies prediction uncertainty and simultaneously encodes local data sparsity, allowing users to identify both unreliable predictions and underrepresented regions of the training distribution in a single diagnostic.
- Predictions for mixtures utilizing novel precursors (e.g., bio-ash) or power levels exceeding 1800 W should be interpreted as preliminary hypotheses requiring laboratory validation.

The intended application scope of this framework is explicitly limited to preliminary experimental screening and mix design optimization within the parameter ranges listed above. It is not a design-grade engineering tool. The framework must not be used for structural design decisions, production quality assurance, or any safety-critical application without independent experimental verification. Predictions generated outside the listed parameter ranges, for underrepresented precursor systems, or for specimens expected to achieve less than 20 MPa should be treated as preliminary hypotheses only. The conformal prediction interval (Figure 13) provides a quantitative indicator of local reliability; half-widths exceeding ±25 MPa explicitly signal that the prediction requires experimental confirmation before use.

## 5. Conclusions

The current study presents a novel stacking ensemble methodology to predict the compressive strength of microwave-cured geopolymers, resulting in excellent predictive performance (*R*^2^ = 0.957). However, a thorough evaluation of the proposed framework reveals several inherent weaknesses that limit its application potential.

The primary limitation of the proposed framework is that all validation (including the Leave-One-Study-Out analysis) is conducted entirely within the literature-derived dataset ecosystem. While LOSO validation ensures no observations from a held-out study appear in the training fold, it does not constitute validation against genuinely independent experimental conditions or industrial-scale microwave curing environments. Its predictive transferability beyond the parameter space of the 21 integrated source studies remains unproven, and final material qualification must always rely on independent laboratory testing. Generating a small independent experimental validation set (even 15–20 specimens from a new laboratory campaign) is identified as the highest-priority next step to establish genuine deployment confidence. Another limitation is the lack of specific chemical descriptors, such as activator modulus and water–binder ratio.

The present framework is a statistical surrogate and does not embed governing relationships such as Maxwell’s equations for dielectric heating or Arrhenius-type activation energy expressions for geopolymerization. Extrapolation outside the investigated parameter space therefore remains unreliable. Consequently, the framework is positioned as a decision-support tool for preliminary experimental screening. It is designed to reduce the trial-and-error space in mix design by identifying physically plausible trends, but final material qualification must still rely on independent laboratory validation. Future work should focus on integrating physics-informed constraints and expanding the dataset through collaborative, standardized experimental campaigns to enhance the model’s extrapolation stability. Moreover, to enhance the scientific depth and extrapolation capabilities beyond the current literature-derived dataset, future iterations of this framework should transition from a purely data-driven surrogate to a physics-informed hybrid model. Key priorities include the integration of dielectric properties (loss tangents), real-time internal temperature profiles, and reaction-kinetic constraints. Proposing such a hybrid architecture will allow the model to capture the complex thermal-gradient-induced stresses and moisture transport mechanisms that govern long-term durability, moving beyond interpolation to true mechanistic prediction.

## Figures and Tables

**Table 1:** Descriptive statistics of the dataset, derived from the 21 integrated experimental studies. A granular study-level breakdown of these variables is provided in Appendix A.

Variable Category	Feature	Mean	SD	Median [IQR]	Min–Max
Material Composition	FA	0.80	0.38	1.00 [0.00]	0.00–1.00
	GGBS	0.16	0.35	0.00 [0.00]	0.00–1.00
	Metakaolin	0.04	0.20	0.00 [0.00]	0.00–1.00
	Silica Fume	0.005	0.01	0.00 [0.00]	0.00–0.10
	Sand/Aggregate	1.43	1.49	1.49 [2.07]	0.00–4.88
Activator Chemistry	NaOH	0.25	0.18	0.22 [0.33]	0.04–0.48
	Molarity (M)	9.26	4.44	10.0 [6.81]	2.00–14.81
	Na_2_SiO_3_	0.14	0.15	0.14 [0.26]	0.00–0.39
Microwave & Geometry	Microwave Power (W)	437.09	403.04	300	0.00–1800.00
	Curing Time (mins)	25.48	29.57	15.0 [35.0]	0.00–120.00
	Specimen Size (mm^2^)	1859.70	921.80	1600	625.00–5041.00
Mechanical State	Testing Age (days)	13.77	32.37	1.0 [13.0]	0.50–180.00
	Compressive Strength (MPa)	34.08	25.68	31.2 [46.1]	1.40–94.10

**Table 2:** RFE feature selection results.

Feature	Rank	Selected
Testing Age (days)	1	Yes
Sand/Aggregate	1	Yes
NaOH	1	Yes
Molarity (M)	1	Yes
Microwave Power (W)	1	Yes
Curing Time (mins)	1	Yes
Specimen Size (mm^2^)	1	Yes
Na_2_SiO_3_	2	Yes
Silica Fume	3	Yes
FA	4	Yes
GGBS	5	Yes
Metakaolin	6	Yes
Waste Glass	7	No
Steel Slag	8	No

**Table 3:** Model performance comparison.

Model	Train *R*^2^	Test *R*^2^	Train RMSE	Test RMSE
Stacking Ensemble (Ridge)	0.9928	0.9571	2.1219	5.6435
XGB	0.9985	0.9554	0.9633	5.7521
LGBM	0.9960	0.9448	1.5822	6.4054
CAT	0.9961	0.9425	1.5717	6.5369
RF	0.9834	0.9276	3.2293	7.3348
ET	0.9994	0.9509	0.5983	6.0401
GBM	0.9981	0.9353	1.0922	6.9331
EBM	0.9845	0.9369	3.1203	6.8446

**Table 4:** Sliced evaluation showing performance across different data segments.

Count	*R* ^2^	RMSE	MAE	Mean Target	Mean Pred
10	0.974	3.093	1.726	28.235	27.277
11	0.720	6.270	5.200	20.607	17.633
6	0.866	6.991	5.552	52.109	51.461
11	0.893	7.120	5.485	33.288	32.308
9	0.984	3.621	3.281	70.024	72.387

**Table 5:** Feature effect analysis for conceptual soundness.

Feature	Min Value	Max Value	Pred @ Min	Pred @ Max	Effect	Direction
Curing Time (mins)	0.000	120.531	10.519	44.138	33.618	↑ Positive
Testing Age (days)	0.000	180.504	20.626	43.929	23.303	↑ Positive
Molarity (M)	1.922	15.020	12.299	26.112	13.812	↑ Positive
Microwave Power (W)	0.000	1806.284	16.099	29.865	13.766	↑ Positive
NaOH	0.039	0.485	27.570	16.090	−11.479	↓ Negative
Sand/Aggregate	0.000	4.911	25.834	14.809	−11.024	↓ Negative
Specimen Size (mm^2^)	601.631	5073.172	31.909	23.554	−8.355	↓ Negative
Metakaolin	0.000	1.000	22.666	17.592	−5.074	↓ Negative
Silica Fume	0.000	0.100	22.666	26.744	4.078	↑ Positive
Na_2_SiO_3_	0.000	0.395	19.634	17.660	−1.974	↓ Negative
FA	0.000	1.015	21.191	22.793	1.603	↑ Positive
GGBS	0.000	1.011	22.633	24.128	1.495	↑ Positive

‘@’ denotes the prediction evaluated at the stated feature value (Min/Max) with all other features held at their dataset median. “↑ = positive directional effect (strength increases with the feature); ↓ = negative directional effect”.

**Table 6:** Leave-One-Study-Out (LOSO) cross-validation performance highlights.

Study Group (Unseen Test Set)	Dataset Size (*n*)	Extrapolation *R*^2^	Extrapolation RMSE (MPa)
High-Volume Studies			
Kwami, 2020 [45]	42	0.985	3.85
Somaratna et al., 2010 [4]	24	0.996	0.99
Tan et al., 2020 [29]	10	0.992	0.48
Moderate-Volume Studies			
Gültekin, 2025 [46]	10	0.94	0.25
Anwar et al., 2025 [26]	16	0.926	1.07
Low-Volume Studies			
Atiku et al., 2023 [31]	4	−172.13	9.44
Mayhoub et al., 2021 [30]	3	−397.11	39.02
Global Median Across All 21 Studies	-	0.703	3.88

## Data Availability

The original contributions presented in this study are included in the article. Further inquiries can be directed to the corresponding author.

## Abbreviations

The following abbreviations are used in this manuscript:

*R*^2^	Coefficient of Determination
RMSE	Root Mean Square Error
OPC	Ordinary Portland Cement
GEP	Gene Expression Programming
ANN	Artificial Neural Network
DNN	Deep Neural Network
GGBFS	Ground Granulated Blast Furnace Slag
GGBS	Ground Granulated Blast Furnace Slag
HTS	High Titanium Slag
RFE	Recursive Feature Elimination
RF	Random Forest
ET	Extra Tree
GBM	Gradient Boosting Machine
XGB	Extreme Gradient Boosting
LGBM	Light Gradient Boosting Machine
GOSS	Gradient-Based One-Side Sampling
CAT	Categorical Boosting Machine
EBM	Explainable Boosting Machine
GAM	Generalized Additive Model
MAE	Mean Absolute Error
MAPE	Mean Absolute Percentage Error
SHAP	Shapley Additive Explanations
GHz	Gigahertz
NaOH	Sodium Hydroxide
Na_2_SiO_3_	Sodium Silicate
FA	Fly Ash
MPa	Megapascals
mins	Minutes
mm^2^	Square Millimeters
W	Watts
IQR	Interquartile Range
SD	Standard Deviation (Microwave Power)
COV	Coefficient of Variation
Min	Minimum
Max	Maximum
UQ	Uncertainty Quantification

## Appendix A. Complete Dataset Used in This Study

The following tables provide a comprehensive overview of the experimental studies integrated into the microwave-curing database. The data represent 248 high-fidelity specimens extracted from peer-reviewed research, grouped by source reference to highlight the chemical and process parameters utilized across different investigations.

**Table A1:** Experimental results dataset from the literature on microwave-cured geopolymers.

Reference	Specimen ID	Precursor Type						Sand/	Activator			Microwave Curing Parameters		Geometry		Testing Age (Days)
		FA	GGBS	Silica Fume	Metakaolin	Waste Glass	Steel Slag	Aggregate	NaOH Molarity (M)	NaOH	Na_2_SiO_3_	Power (W)	Curing Time (mins)	Cube Edge Length (mm)	Size of Specimen (mm^2^)	
Graytee et al., 2018 [6]	G-1	1	0	0	0	0	0	0	8	0.0714	0.1786	200	5	50	2500	1
	G-2	1	0	0	0	0	0	0	8	0.0714	0.1786	200	10	50	2500	1
	G-3	1	0	0	0	0	0	0	8	0.0714	0.1786	200	15	50	2500	1
	G-4	1	0	0	0	0	0	0	8	0.0714	0.1786	200	20	50	2500	1
	G-5	1	0	0	0	0	0	0	8	0.0714	0.1786	200	25	50	2500	1
	G-6	1	0	0	0	0	0	0	8	0.0714	0.1786	200	30	50	2500	1
	G-7	1	0	0	0	0	0	0	8	0.0714	0.1786	200	45	50	2500	1
	G-8	1	0	0	0	0	0	0	8	0.0714	0.1786	200	60	50	2500	1
	G-9	1	0	0	0	0	0	0	8	0.0714	0.1786	300	15	50	2500	1
	G-10	1	0	0	0	0	0	0	8	0.0714	0.1786	400	15	50	2500	1
	G-11	1	0	0	0	0	0	0	8	0.0714	0.1786	600	13	50	2500	1
Graytee et al., 2018 [6]	G-12	1	0	0	0	0	0	0	8	0.0714	0.1786	200	5	25	625	1
	G-13	1	0	0	0	0	0	0	8	0.0714	0.1786	200	10	25	625	1
	G-14	1	0	0	0	0	0	0	8	0.0714	0.1786	200	15	25	625	1
Chindaprasirt et al., 2013 [3]	C-15	1	0	0	0	0	0	1.5	10	0.2154	0.3231	0	0	50	2500	7
	C-16	1	0	0	0	0	0	1.5	10	0.2154	0.3231	0	0	50	2500	28
	C-17	1	0	0	0	0	0	1.5	10	0.2154	0.3231	90	3	50	2500	7
	C-18	1	0	0	0	0	0	1.5	10	0.2154	0.3231	90	5	50	2500	7
	C-19	1	0	0	0	0	0	1.5	10	0.2154	0.3231	90	10	50	2500	7
Somaratna et al., 2010 [4]	S-20	1	0	0	0	0	0	2.0752	4	0.4	0	240	30	50	2500	1
	S-21	1	0	0	0	0	0	2.0752	4	0.4	0	240	45	50	2500	1
	S-22	1	0	0	0	0	0	2.0752	4	0.4	0	240	60	50	2500	1
	S-23	1	0	0	0	0	0	2.0752	4	0.4	0	240	75	50	2500	1
	S-24	1	0	0	0	0	0	2.0752	4	0.4	0	240	90	50	2500	1
	S-25	1	0	0	0	0	0	2.0752	4	0.4	0	240	120	50	2500	1
	S-26	1	0	0	0	0	0	2.0752	6	0.4	0	240	30	50	2500	1
	S-27	1	0	0	0	0	0	2.0752	6	0.4	0	240	45	50	2500	1
	S-28	1	0	0	0	0	0	2.0752	6	0.4	0	240	60	50	2500	1
	S-29	1	0	0	0	0	0	2.0752	6	0.4	0	240	75	50	2500	1
	S-30	1	0	0	0	0	0	2.0752	6	0.4	0	240	90	50	2500	1
	S-31	1	0	0	0	0	0	2.0752	6	0.4	0	240	120	50	2500	1
	S-32	1	0	0	0	0	0	2.0752	8	0.4	0	240	30	50	2500	1
	S-33	1	0	0	0	0	0	2.0752	8	0.4	0	240	45	50	2500	1
	S-34	1	0	0	0	0	0	2.0752	8	0.4	0	240	60	50	2500	1
	S-35	1	0	0	0	0	0	2.0752	8	0.4	0	240	75	50	2500	1
	S-36	1	0	0	0	0	0	2.0752	8	0.4	0	240	90	50	2500	1
	S-37	1	0	0	0	0	0	2.0752	8	0.4	0	240	120	50	2500	1
	S-38	1	0	0	0	0	0	2.0752	10	0.4	0	240	30	50	2500	1
	S-39	1	0	0	0	0	0	2.0752	10	0.4	0	240	45	50	2500	1
	S-40	1	0	0	0	0	0	2.0752	10	0.4	0	240	60	50	2500	1
	S-41	1	0	0	0	0	0	2.0752	10	0.4	0	240	75	50	2500	1
	S-42	1	0	0	0	0	0	2.0752	10	0.4	0	240	90	50	2500	1
	S-43	1	0	0	0	0	0	2.0752	10	0.4	0	240	120	50	2500	1
Mohsen et al., 2021 [24]	M-44	0	1	0	0	0	0	0	5	0.06	0.12	0	0	25.4	645.16	1
	M-45	0	1	0	0	0	0	0	5	0.06	0.12	0	0	25.4	645.16	7
	M-46	0	1	0	0	0	0	0	5	0.06	0.12	0	0	25.4	645.16	28
	M-47	0	1	0	0	0	0	0	5	0.06	0.12	90	2	25.4	645.16	1
	M-48	0	1	0	0	0	0	0	5	0.06	0.12	450	2	25.4	645.16	1
	M-49	0	1	0	0	0	0	0	5	0.06	0.12	900	2	25.4	645.16	1
	M-50	0	1	0	0	0	0	0	5	0.06	0.12	90	2	25.4	645.16	7
	M-51	0	1	0	0	0	0	0	5	0.06	0.12	450	2	25.4	645.16	7
	M-52	0	1	0	0	0	0	0	5	0.06	0.12	900	2	25.4	645.16	7
	M-53	0	1	0	0	0	0	0	5	0.06	0.12	90	2	25.4	645.16	28
	M-54	0	1	0	0	0	0	0	5	0.06	0.12	450	2	25.4	645.16	28
	M-55	0	1	0	0	0	0	0	5	0.06	0.12	900	2	25.4	645.16	28
	M-56	0	1	0	0	0	0	0	5	0.06	0.12	90	4	25.4	645.16	1
	M-57	0	1	0	0	0	0	0	5	0.06	0.12	450	4	25.4	645.16	1
	M-58	0	1	0	0	0	0	0	5	0.06	0.12	900	4	25.4	645.16	1
	M-59	0	1	0	0	0	0	0	5	0.06	0.12	90	4	25.4	645.16	7
	M-60	0	1	0	0	0	0	0	5	0.06	0.12	450	4	25.4	645.16	7
	M-61	0	1	0	0	0	0	0	5	0.06	0.12	900	4	25.4	645.16	7
	M-62	0	1	0	0	0	0	0	5	0.06	0.12	90	4	25.4	645.16	28
	M-63	0	1	0	0	0	0	0	5	0.06	0.12	450	4	25.4	645.16	28
	M-64	0	1	0	0	0	0	0	5	0.06	0.12	900	4	25.4	645.16	28
	M-65	0	1	0	0	0	0	0	5	0.06	0.12	90	8	25.4	645.16	1
	M-66	0	1	0	0	0	0	0	5	0.06	0.12	450	8	25.4	645.16	1
	M-67	0	1	0	0	0	0	0	5	0.06	0.12	900	8	25.4	645.16	1
	M-68	0	1	0	0	0	0	0	5	0.06	0.12	90	8	25.4	645.16	7
	M-69	0	1	0	0	0	0	0	5	0.06	0.12	450	8	25.4	645.16	7
	M-70	0	1	0	0	0	0	0	5	0.06	0.12	900	8	25.4	645.16	7
	M-71	0	1	0	0	0	0	0	5	0.06	0.12	90	8	25.4	645.16	28
	M-72	0	1	0	0	0	0	0	5	0.06	0.12	450	8	25.4	645.16	28
	M-73	0	1	0	0	0	0	0	5	0.06	0.12	900	8	25.4	645.16	28
Khaleel et al., 2021 [20]	Kh-74	1	0	0	0	0	0	3	14.81	0.478	0	0	0	40	1600	1
	Kh-75	1	0	0	0	0	0	3	14.81	0.478	0	100	5	40	1600	1
	Kh-76	1	0	0	0	0	0	3	14.81	0.478	0	100	10	40	1600	1
	Kh-77	1	0	0	0	0	0	3	14.81	0.478	0	100	15	40	1600	1
	Kh-78	1	0	0	0	0	0	3	14.81	0.478	0	100	30	40	1600	1
	Kh-79	1	0	0	0	0	0	3	14.81	0.478	0	100	45	40	1600	1
	Kh-80	1	0	0	0	0	0	3	14.81	0.478	0	100	60	40	1600	1
	Kh-81	1	0	0	0	0	0	3	14.81	0.478	0	180	5	40	1600	1
	Kh-82	1	0	0	0	0	0	3	14.81	0.478	0	180	10	40	1600	1
	Kh-83	1	0	0	0	0	0	3	14.81	0.478	0	180	15	40	1600	1
	Kh-84	1	0	0	0	0	0	3	14.81	0.478	0	180	30	40	1600	1
	Kh-85	1	0	0	0	0	0	3	14.81	0.478	0	180	45	40	1600	1
	Kh-86	1	0	0	0	0	0	3	14.81	0.478	0	180	60	40	1600	1
	Kh-87	1	0	0	0	0	0	3	14.81	0.478	0	300	5	40	1600	1
	Kh-88	1	0	0	0	0	0	3	14.81	0.478	0	300	10	40	1600	1
	Kh-89	1	0	0	0	0	0	3	14.81	0.478	0	300	15	40	1600	1
	Kh-90	1	0	0	0	0	0	3	14.81	0.478	0	300	30	40	1600	1
Kwami, 2020 [45]	K-91	1	0	0	0	0	0	3	14.81	0.478	0	180	15	40	1600	1
	K-92	1	0	0	0	0	0	3	14.81	0.478	0	180	30	40	1600	1
	K-93	1	0	0	0	0	0	3	14.81	0.478	0	180	45	40	1600	1
	K-94	1	0	0	0	0	0	3	14.81	0.478	0	180	60	40	1600	1
	K-95	1	0	0	0	0	0	3	14.81	0.478	0	180	75	40	1600	1
	K-96	1	0	0	0	0	0	3	14.81	0.478	0	180	90	40	1600	1
	K-97	1	0	0	0	0	0	3	14.81	0.478	0	180	15	40	1600	3
	K-98	1	0	0	0	0	0	3	14.81	0.478	0	180	30	40	1600	3
	K-99	1	0	0	0	0	0	3	14.81	0.478	0	180	45	40	1600	3
	K-100	1	0	0	0	0	0	3	14.81	0.478	0	180	60	40	1600	3
	K-101	1	0	0	0	0	0	3	14.81	0.478	0	180	75	40	1600	3
	K-102	1	0	0	0	0	0	3	14.81	0.478	0	180	90	40	1600	3
	K-103	1	0	0	0	0	0	3	14.81	0.478	0	180	15	40	1600	7
	K-104	1	0	0	0	0	0	3	14.81	0.478	0	180	30	40	1600	7
	K-105	1	0	0	0	0	0	3	14.81	0.478	0	180	45	40	1600	7
	K-106	1	0	0	0	0	0	3	14.81	0.478	0	180	60	40	1600	7
	K-107	1	0	0	0	0	0	3	14.81	0.478	0	180	75	40	1600	7
	K-108	1	0	0	0	0	0	3	14.81	0.478	0	180	90	40	1600	7
	K-109	1	0	0	0	0	0	3	14.81	0.478	0	180	15	40	1600	28
	K-110	1	0	0	0	0	0	3	14.81	0.478	0	180	30	40	1600	28
	K-111	1	0	0	0	0	0	3	14.81	0.478	0	180	45	40	1600	28
	K-112	1	0	0	0	0	0	3	14.81	0.478	0	180	60	40	1600	28
	K-113	1	0	0	0	0	0	3	14.81	0.478	0	180	75	40	1600	28
	K-114	1	0	0	0	0	0	3	14.81	0.478	0	180	90	40	1600	28
	K-115	1	0	0	0	0	0	3	14.81	0.478	0	180	15	40	1600	56
	K-116	1	0	0	0	0	0	3	14.81	0.478	0	180	30	40	1600	56
	K-117	1	0	0	0	0	0	3	14.81	0.478	0	180	45	40	1600	56
	K-118	1	0	0	0	0	0	3	14.81	0.478	0	180	60	40	1600	56
	K-119	1	0	0	0	0	0	3	14.81	0.478	0	180	75	40	1600	56
	K-120	1	0	0	0	0	0	3	14.81	0.478	0	180	90	40	1600	56
	K-121	1	0	0	0	0	0	3	14.81	0.478	0	180	15	40	1600	90
	K-122	1	0	0	0	0	0	3	14.81	0.478	0	180	30	40	1600	90
	K-123	1	0	0	0	0	0	3	14.81	0.478	0	180	45	40	1600	90
	K-124	1	0	0	0	0	0	3	14.81	0.478	0	180	60	40	1600	90
	K-125	1	0	0	0	0	0	3	14.81	0.478	0	180	75	40	1600	90
	K-126	1	0	0	0	0	0	3	14.81	0.478	0	180	90	40	1600	90
	K-127	1	0	0	0	0	0	3	14.81	0.478	0	180	15	40	1600	180
	K-128	1	0	0	0	0	0	3	14.81	0.478	0	180	30	40	1600	180
	K-129	1	0	0	0	0	0	3	14.81	0.478	0	180	45	40	1600	180
	K-130	1	0	0	0	0	0	3	14.81	0.478	0	180	60	40	1600	180
	K-131	1	0	0	0	0	0	3	14.81	0.478	0	180	75	40	1600	180
	K-132	1	0	0	0	0	0	3	14.81	0.478	0	180	90	40	1600	180
Hong and Kim, 2019 [23]	H-133	1	0	0	0	0	0	0	14	0.38	0	700	0	50	2500	0.5
	H-134	1	0	0	0	0	0	0	14	0.38	0	700	1	50	2500	0.5
	H-135	1	0	0	0	0	0	0	14	0.38	0	700	2	50	2500	0.5
	H-136	1	0	0	0	0	0	0	14	0.38	0	700	3	50	2500	0.5
	H-137	1	0	0	0	0	0	0	14	0.38	0	700	4	50	2500	0.5
	H-138	1	0	0	0	0	0	0	14	0.38	0	700	5	50	2500	0.5
	H-139	1	0	0	0	0	0	0	14	0.38	0	700	6	50	2500	0.5
	H-140	1	0	0	0	0	0	0	14	0.38	0	700	7	50	2500	0.5
	H-141	1	0	0	0	0	0	0	14	0.38	0	700	0	50	2500	1
	H-142	1	0	0	0	0	0	0	14	0.38	0	700	1	50	2500	1
	H-143	1	0	0	0	0	0	0	14	0.38	0	700	2	50	2500	1
	H-144	1	0	0	0	0	0	0	14	0.38	0	700	3	50	2500	1
	H-145	1	0	0	0	0	0	0	14	0.38	0	700	4	50	2500	1
	H-146	1	0	0	0	0	0	0	14	0.38	0	700	5	50	2500	1
	H-147	1	0	0	0	0	0	0	14	0.38	0	700	0	50	2500	1.5
	H-148	1	0	0	0	0	0	0	14	0.38	0	700	1	50	2500	1.5
	H-149	1	0	0	0	0	0	0	14	0.38	0	700	2	50	2500	1.5
	H-150	1	0	0	0	0	0	0	14	0.38	0	700	3	50	2500	1.5
	H-151	1	0	0	0	0	0	0	14	0.38	0	700	4	50	2500	1.5
	H-152	1	0	0	0	0	0	0	14	0.38	0	700	5	50	2500	1.5
Chindaprasirt et al., 2013 [28]	Ch-153	1	0	0	0	0	0	1.5	10	0.14	0.21	0	0	50	2500	7
	Ch-154	1	0	0	0	0	0	1.5	10	0.14	0.21	0	0	50	2500	7
	Ch-155	1	0	0	0	0	0	1.5	10	0.14	0.21	90	5	50	2500	7
	Ch-156	1	0	0	0	0	0	1.5	10	0.14	0.21	90	5	50	2500	7
	Ch-157	1	0	0	0	0	0	1.5	10	0.14	0.21	90	5	50	2500	7
Kastiukas et al., 2020 [19]	Ka-158	0	1	0	0	0	0	2.75	12.821	0.0975	0.3925	350	5	40	1600	3
	Ka-159	0	1	0	0	0	0	2.75	12.821	0.0975	0.3925	540	5	40	1600	3
	Ka-160	0	1	0	0	0	0	2.75	12.821	0.0975	0.3925	750	5	40	1600	3
Tan et al., 2020 [29]	T-161	0	0	0	1	0	0	0	10	0.1600	0.2400	0	0	40	1600	3
	T-162	0	0	0	1	0	0	0	10	0.1600	0.2400	1000	20	40	1600	3
	T-163	0	0	0	1	0	0	0	10	0.1600	0.2400	1000	40	40	1600	3
	T-164	0	0	0	1	0	0	0	10	0.1600	0.2400	1000	60	40	1600	3
	T-165	0	0	0	1	0	0	0	10	0.1600	0.2400	1000	80	40	1600	3
	T-166	0	0	0	1	0	0	0	10	0.1600	0.2400	1000	40	40	1600	1
	T-167	0	0	0	1	0	0	0	10	0.1600	0.2400	1000	40	40	1600	3
	T-168	0	0	0	1	0	0	0	10	0.1600	0.2400	1000	40	40	1600	5
	T-169	0	0	0	1	0	0	0	10	0.1600	0.2400	1000	40	40	1600	7
	T-170	0	0	0	1	0	0	0	10	0.1600	0.2400	1000	40	40	1600	9
Suwan et al., 2021 [17]	Su-171	1	0	0	0	0	0	2.75	10	0.2400	0.3600	450	5	40	1600	3
	Su-172	1	0	0	0	0	0	2.75	10	0.2400	0.3600	450	5	40	1600	28
Mayhoub et al., 2021 [30]	Ma-173	0	0.7614	0.2386	0	0	0	0.8985	12	0.1143	0.2857	720	4	50	2500	3
	Ma-174	0	0.7614	0.2386	0	0	0	0.8985	12	0.1143	0.2857	720	4	50	2500	7
	Ma-175	0	0.7614	0.2386	0	0	0	0.8985	12	0.1143	0.2857	720	4	50	2500	28
Watanabe et al., 2023 [42]	W-176	1	0	0	0	0	0	0	2	0.1544	0.3861	300	1	25	625	7
	W-177	1	0	0	0	0	0	0	2	0.1544	0.3861	700	1	25	625	7
	W-178	1	0	0	0	0	0	0	6	0.1544	0.3861	300	1	25	625	7
	W-179	1	0	0	0	0	0	0	6	0.1544	0.3861	700	1	25	625	7
	W-180	1	0	0	0	0	0	0	10	0.1544	0.3861	300	1	25	625	7
	W-181	1	0	0	0	0	0	0	10	0.1544	0.3861	700	1	25	625	7
Atiku et al., 2023 [31]	A-182	1	0	0	0	0	0	0	0.1	0.2222	0.4444	0	0	50	2500	14
	A-183	1	0	0	0	0	0	0	0.1	0.2222	0.4444	200	10	50	2500	14
	A-184	1	0	0	0	0	0	0	0.1	0.2222	0.4444	200	15	50	2500	14
	A-185	1	0	0	0	0	0	0	0.1	0.2222	0.4444	200	20	50	2500	14
Polat, 2023 [21]	P-186	0.9	0	0.1	0	0	0	3	12	0.1667	0.3333	200	10	50	2500	7
	P-187	0.9	0	0.1	0	0	0	3	12	0.1667	0.3333	300	10	50	2500	7
	P-188	0.9	0	0.1	0	0	0	3	12	0.1667	0.3333	400	10	50	2500	7
	P-189	0.9	0	0.1	0	0	0	3	12	0.1667	0.3333	500	10	50	2500	7
	P-190	0.9	0	0.1	0	0	0	3	12	0.1667	0.3333	600	10	50	2500	7
Guan, 2023 [44]	Gu-191	1	0	0	0	0	0	0	5.6452	0.0766	0.3064	119	12	40	1600	1
	Gu-192	1	0	0	0	0	0	0	5.6452	0.0766	0.3064	119	24	40	1600	1
	Gu-193	1	0	0	0	0	0	0	5.6452	0.0766	0.3064	119	48	40	1600	1
	Gu-194	1	0	0	0	0	0	0	5.6452	0.0766	0.3064	119	72	40	1600	1
	Gu-195	1	0	0	0	0	0	0	5.6452	0.0766	0.3064	119	84	40	1600	1
	Gu-196	1	0	0	0	0	0	0	5.6452	0.0766	0.3064	231	5	40	1600	1
	Gu-197	1	0	0	0	0	0	0	5.6452	0.0766	0.3064	231	12	40	1600	1
	Gu-198	1	0	0	0	0	0	0	5.6452	0.0766	0.3064	231	18	40	1600	1
	Gu-199	1	0	0	0	0	0	0	5.6452	0.0766	0.3064	231	24	40	1600	1
	Gu-200	1	0	0	0	0	0	0	5.6452	0.0766	0.3064	231	30	40	1600	1
	Gu-201	1	0	0	0	0	0	0	5.6452	0.0766	0.3064	231	36	40	1600	1
	Gu-202	1	0	0	0	0	0	0	5.6452	0.0766	0.3064	231	42	40	1600	1
	Gu-203	1	0	0	0	0	0	0	5.6452	0.0766	0.3064	385	4	40	1600	1
	Gu-204	1	0	0	0	0	0	0	5.6452	0.0766	0.3064	385	8	40	1600	1
	Gu-205	1	0	0	0	0	0	0	5.6452	0.0766	0.3064	385	12	40	1600	1
	Gu-206	1	0	0	0	0	0	0	5.6452	0.0766	0.3064	385	16	40	1600	1
	Gu-207	1	0	0	0	0	0	0	5.6452	0.0766	0.3064	385	20	40	1600	1
	Gu-208	1	0	0	0	0	0	0	5.6452	0.0766	0.3064	385	24	40	1600	1
	Gu-209	1	0	0	0	0	0	0	5.6452	0.0766	0.3064	539	3	40	1600	1
	Gu-210	1	0	0	0	0	0	0	5.6452	0.0766	0.3064	539	6	40	1600	1
	Gu-211	1	0	0	0	0	0	0	5.6452	0.0766	0.3064	539	9	40	1600	1
	Gu-212	1	0	0	0	0	0	0	5.6452	0.0766	0.3064	539	12	40	1600	1
	Gu-213	1	0	0	0	0	0	0	5.6452	0.0766	0.3064	539	15	40	1600	1
Gultekin, 2024 [43]	Gul-214	0	0	0	1	0	0	3	3	0.0533	0.4197	300	30	40	1600	1
	Gul-215	0	0	0	1	0	0	3	3	0.0533	0.4197	300	30	40	1600	1
Carollo et al., 2025 [47]	Ca-216	0	0	0	0	1	0	0	2.5	0.5	0.0000	0	0	10	100	7
	Ca-217	0	0	0	0	1	0	0	2.5	0.5	0.0000	450	5	10	100	1
	Ca-218	0	0	0	0	1	0	0	5	0.5	0.0000	450	5	10	100	1
Gültekin, 2025 [46]	Gül-219	1	0	0	0	0	0	4.8845	3.7	0.0429	0.3338	0	0	71	5041	1
	Gül-220	1	0	0	0	0	0	4.8845	3.7	0.0429	0.3338	460	5	71	5041	1
	Gül-221	1	0	0	0	0	0	4.8845	3.7	0.0429	0.3338	460	10	71	5041	1
	Gül-222	1	0	0	0	0	0	4.8845	3.7	0.0429	0.3338	460	15	71	5041	1
	Gül-223	1	0	0	0	0	0	4.8845	3.7	0.0429	0.3338	600	5	71	5041	1
	Gül-224	1	0	0	0	0	0	4.8845	3.7	0.0429	0.3338	600	10	71	5041	1
	Gül-225	1	0	0	0	0	0	4.8845	3.7	0.0429	0.3338	600	15	71	5041	1
	Gül-226	1	0	0	0	0	0	4.8845	3.7	0.0429	0.3338	700	5	71	5041	1
	Gül-227	1	0	0	0	0	0	4.8845	3.7	0.0429	0.3338	700	10	71	5041	1
	Gül-228	1	0	0	0	0	0	4.8845	3.7	0.0429	0.3338	700	15	71	5041	1
Anwar et al., 2025 [26]	An-229	0.7	0.3	0	0	0	0	0.8	2.9	0.0780	0.3538	1260	0	40	1600	7
	An-230	0.7	0.3	0	0	0	0	0.8	2.9	0.0780	0.3539	1260	0	40	1600	28
	An-231	0.7	0.3	0	0	0	0	0.8	2.9	0.0780	0.3539	1260	0.25	40	1600	7
	An-232	0.7	0.3	0	0	0	0	0.8	2.9	0.0780	0.3539	1260	0.25	40	1600	28
	An-233	0.7	0.3	0	0	0	0	0.8	2.9	0.0780	0.3539	1260	0.5	40	1600	7
	An-234	0.7	0.3	0	0	0	0	0.8	2.9	0.0780	0.3539	1260	0.5	40	1600	28
	An-235	0.7	0.3	0	0	0	0	0.8	2.9	0.0780	0.3539	1260	0.75	40	1600	7
	An-236	0.7	0.3	0	0	0	0	0.8	2.9	0.0780	0.3539	1260	0.75	40	1600	28
	An-237	0.7	0.3	0	0	0	0	0.8	2.9	0.0780	0.3539	1800	0	40	1600	7
	An-238	0.7	0.3	0	0	0	0	0.8	2.9	0.0780	0.3539	1800	0	40	1600	28
	An-239	0.7	0.3	0	0	0	0	0.8	2.9	0.0780	0.3539	1800	0.25	40	1600	7
	An-240	0.7	0.3	0	0	0	0	0.8	2.9	0.0780	0.3539	1800	0.25	40	1600	28
	An-241	0.7	0.3	0	0	0	0	0.8	2.9	0.0780	0.3539	1800	0.5	40	1600	7
	An-242	0.7	0.3	0	0	0	0	0.8	2.9	0.0780	0.3539	1800	0.5	40	1600	28
	An-243	0.7	0.3	0	0	0	0	0.8	2.9	0.0780	0.3539	1800	0.75	40	1600	7
	An-244	0.7	0.3	0	0	0	0	0.8	2.9	0.0780	0.3539	1800	0.75	40	1600	28
He et al., 2025 [48]	He-245	0.3	0	0	0	0	0.7	0	1.7	0.0230	0.1437	0	0	40	1600	7
	He-246	0.3	0	0	0	0	0.7	0	1.7	0.0230	0.1437	0	0	40	1600	28
	He-247	0.3	0	0	0	0	0.7	0	1.7	0.0230	0.1437	70	16	40	1600	7
	He-248	0.3	0	0	0	0	0.7	0	1.7	0.0230	0.1437	70	16	40	1600	28

**Table A2:** Summary of experimental studies included in the compiled database.

Reference	Precursor Type	NaOH Molarity (M)	Microwave Power (W)	Microwave Curing Time (mins)	Compressive Strength (MPa)	Testing Age (Days)	No. of Specimens	No. of Excluded Observations	Reason for Exclusion
Graytee et al., 2018 [6]	FA	8	200.0–600.0	5.0–60.0	23.00–90.00	1	14	0	N/A
Chindaprasirt et al., 2013 [3]	FA	10	0.0–90.0	0.0–10.0	9.40–35.80	7.0–28.0	5	0	N/A
Somaratna et al., 2010 [4]	FA	4.00–10.00	240	30.0–120.0	6.41–56.87	1	24	0	N/A
Mohsen et al., 2021 [24]	GGBS	5	0.0–900.0	0.0–8.0	3.30–31.23	1.0–28.0	30	0	N/A
Khaleel et al., 2021 [20]	FA	14.81	0.0–300.0	0.0–60.0	5.40–77.00	1	17	0	N/A
Kwami, 2020 [45]	FA	14.81	180	15.0–90.0	7.90–94.10	1.0–180.0	42	0	N/A
Hong and Kim, 2019 [23]	FA	14	700	0.0–7.0	1.40–41.50	0.5–1.5	20	0	N/A
Chindaprasirt et al., 2013 [28]	FA	10	0.0–900.0	0.0–5.0	20.0–42.50	7	5	0	N/A
Kastiukas et al., 2020 [19]	GGBS	12.82	350.0–750.0	5	28.24–43.08	3	3	1	Missing essential mix descriptors
Tan et al., 2020 [29]	Metakaolin	10	0.0–1000.0	0.0–80.0	8.39–26.90	1.0–9.0	10	1	Ambiguous unit reporting that could not be harmonized
Suwan et al., 2021 [17]	FA	10	450	5	3.30–4.98	3.0–28.0	2	0	N/A
Mayhoub et al., 2021 [30]	GGBS/Silica Fume	12	720	4	75.25–80.00	3.0–28.0	3	2	Ambiguous unit reporting that could not be harmonized
Watanabe et al., 2023 [42]	FA	2.00–10.00	300.0–700.0	1	7.79–24.72	7	6	0	N/A
Atiku et al., 2023 [31]	FA	0.1	0.0–200.0	0.0–20.0	5.19–7.14	14	4	0	N/A
Polat, 2023 [21]	FA/Silica Fume	12	200.0–600.0	10	39.50–67.70	7	5	0	N/A
Guan, 2023 [44]	FA	5.65	119.0–539.0	3.0–84.0	2.94–37.05	1	23	0	N/A
Gultekin, 2024 [43]	Metakaolin	3	300	30	33.50–40.10	1	2	2	Physical inconsistency (statistical anomaly flagged via Isolation Forest lacking heuristic verification)
Carollo et al., 2025 [47]	Waste Glass	2.50–5.00	0.0–450.0	0.0–5.0	10.30–35.00	1.0–7.0	3	3	Missing essential mix descriptors (e.g., unreported specimen geometry or activator concentration)
Gültekin, 2025 [46]	FA	3.7	0.0–700.0	0.0–15.0	8.20–12.20	1	10	0	N/A
Anwar et al., 2025 [26]	FA/GGBS	2.9	1260.0–1800.0	0.0–0.75	43.61–56.08	7.0–28.0	16	0	N/A
He et al., 2025 [48]	FA/Steel Slag	1.7	0.0–70.0	0.0–16.0	8.50–28.00	7.0–28.0	4	4	Physical inconsistency (reported strengths contradicted geopolymerization kinetics for the given microwave power)

Notes: Microwave Power and Time ranges account for both cured specimens and control samples (where Power/Time = 0) present within the same study. Values are presented as ranges where applicable. N/A: Not excluded.

## Appendix B. Overfitting Diagnostics

This appendix provides supplementary diagnostic evaluations to confirm the robustness and generalization capacity of the proposed stacking ensemble framework. Specifically, learning curves are presented to track the convergence of training and validation errors as a function of dataset size, ensuring the model mitigates high variance. Additionally, a regularization sensitivity analysis is provided for the Ridge meta-learner to demonstrate optimal bias-variance balancing across varying penalty thresholds.
